# Identifying epigenetic biomarkers of established prognostic factors and survival in a clinical cohort of individuals with oropharyngeal cancer

**DOI:** 10.1186/s13148-020-00870-0

**Published:** 2020-06-29

**Authors:** Ryan Langdon, Rebecca Richmond, Hannah R. Elliott, Tom Dudding, Nabila Kazmi, Chris Penfold, Kate Ingarfield, Karen Ho, Andrew Bretherick, Chris Haley, Yanni Zeng, Rosie M. Walker, Michael Pawlita, Tim Waterboer, Tom Gaunt, George Davey Smith, Matthew Suderman, Steve Thomas, Andy Ness, Caroline Relton

**Affiliations:** 1 MRC Integrative Epidemiology Unit at the University of Bristol, Bristol, UK; 2 Population Health Sciences, Bristol Medical School, University of Bristol, Bristol, UK; 3 NIHR Bristol Biomedical Research Centre, University Hospitals Bristol and University of Bristol, Bristol, UK; 4 MRC Human Genetics Unit, Institute of Genetics and Molecular Medicine, University of Edinburgh, Western General Hospital, Crewe Road, Scotland Bristol, EH4 2XU UK; 5 Faculty of Forensic Medicine, Zhongshan School of Medicine, Sun Yat-Sen University, Guangzhou, China; 6 Guangdong Province Translational Forensic Medicine Engineering Technology Research Center, Zhongshan School of Medicine, Sun Yat-Sen University, Guangzhou, China; 7 Medical Genetics Section, Centre for Genomic and Experimental Medicine, Institute of Genetics and Molecular Medicine, University of Edinburgh, Edinburgh, EH4 2XU UK; 8 Centre for Cognitive Ageing and Cognitive Epidemiology, University of Edinburgh, Edinburgh, EH8 9JZ UK; 9 Infections and Cancer Epidemiology, German Cancer Research Center (DKFZ), Heidelberg, Germany

## Abstract

**Background:**

Smoking status, alcohol consumption and HPV infection (acquired through sexual activity) are the predominant risk factors for oropharyngeal cancer and are thought to alter the prognosis of the disease. Here, we conducted single-site and differentially methylated region (DMR) epigenome-wide association studies (EWAS) of these factors, in addition to ∼ 3-year survival, using Illumina Methylation EPIC DNA methylation profiles from whole blood in 409 individuals as part of the Head and Neck 5000 (HN5000) study. Overlapping sites between each factor and survival were then assessed using two-step Mendelian randomization to assess whether methylation at these positions causally affected survival.

**Results:**

Using the MethylationEPIC array in an OPC dataset, we found novel CpG associations with smoking, alcohol consumption and ~ 3-year survival. We found no CpG associations below our multiple testing threshold associated with HPV16 E6 serological response (used as a proxy for HPV infection). CpG site associations below our multiple-testing threshold (*P*_Bonferroni_ < 0.05) for both a prognostic factor and survival were observed at four gene regions: *SPEG* (smoking), *GFI1* (smoking), *PPT2* (smoking) and *KHDC3L* (alcohol consumption). Evidence for a causal effect of DNA methylation on survival was only observed in the *SPEG* gene region (HR per SD increase in methylation score 1.28, 95% CI 1.14 to 1.43, *P* 2.12 × 10^−05^).

**Conclusions:**

Part of the effect of smoking on survival in those with oropharyngeal cancer may be mediated by methylation at the *SPEG* gene locus. Replication in data from independent datasets and data from HN5000 with longer follow-up times is needed to confirm these findings.

## Introduction

Head and neck cancer (HNC) is the eighth most commonly diagnosed type of cancer, with over 12,000 new cases diagnosed in the UK in 2015 [1]. Recently, oropharyngeal cancer (OPC), a subtype of HNC, has shown a significant increase in incidence in the UK. It has more than doubled between 1990 and 2006, with a further doubling since 2010 [2] and is affecting younger (< 45 years old) populations with greater frequency [3]. OPC shows poor survival rates, with the 5-year relative survival rate for the more recently diagnosed oropharyngeal cases (between 2009 and 2013) estimated to be around 55–60% [4].

Several lifestyle and dietary factors as well as viral infections have been implicated in altering both incidence and prognosis for OPC [5–7]. Of particular importance for both incidence [5, 8, 9] and prognosis of OPC [10] are smoking, alcohol intake and HPV type 16 infection (via sexual contact, including that of oral sex). Smoking and, to a lesser extent, heavy drinking at the time of diagnosis are both associated with increased incidence and poor prognosis [10–12]. Interestingly, HPV16 infection, while being a risk factor for OPC incidence, is associated with improved prognosis [13–15]. One study showed improved overall radically improved 4-year survival for HPV-driven OPC (HR, 0.1; 95% CI 0.02–0.4; *N*, 448) [16].

DNA methylation signatures may also serve as valuable prognostic markers for cancer and can be measured using rapid high-throughput approaches [17]. While several whole-genome methylation assays have been performed to define the DNA methylation signature of tumour samples [18, 19], the ability to study cancers through non-invasive sampling of body fluids is a rapidly advancing development in cancer diagnostics and prognosis. In particular, biomarkers identified in blood hold promise as non-invasive prognostic tools and may potentially be used to direct treatment if shown to be informative proxies for cancer development and prognosis [20].

Ultimately, smoking, alcohol consumption and HPV16 infection may influence DNA methylation patterns which therefore have potential as novel exposure or prognostic indicators in OPC [21–23]. Furthermore, as epigenetic changes are a hallmark process of cancer [24], DNA methylation patterns associated with cancer survival may provide insight into biologically relevant pathways. More specifically, these epigenetic changes may act as intermediates on the pathways by which exposures influence survival. For example, as viral infections are thought to play an important role in altering epigenetic processes [25–27], these may serve as a mechanism by which having a HPV16 infection might confer a protective effect over not having one. However, distinguishing a causal mediating role of these epigenetic changes from other explanations such as confounding and reverse causation is challenging and requires more advanced methodological techniques, including the use of Mendelian randomization (MR) [28–30]. MR is an approach which uses genetic variants strongly associated with modifiable exposures to appraise the causal effect of the exposures on disease risk. This approach has been extended to interrogate the causal relationship with molecular intermediates such as DNA methylation [29, 30].

In the setting of a large prospective head and neck cancer cohort (the Head and Neck 5000 Study), we profiled DNA methylation from whole blood in 443 participants with oropharyngeal cancer close to time of diagnosis and prior to treatment starting. We aimed to perform epigenome-wide association analyses (single-site EWAS and differentially methylated region [DMR] analysis) of the main prognostic factors for oropharyngeal cancer (alcohol, smoking and HPV16 infection) as well as survival up to ~ 3 years. We then assessed overlap between the DNA methylation profiles related to these prognostic factors and survival. Where there was evidence of a shared signal, we performed Mendelian randomization analysis to appraise the causal effect of DNA methylation in mediating the effect of these factors on survival.

## Results

Baseline characteristics of samples with epigenetic data, compared to the wider HNC and OPC samples in HN5000 are shown in Table 1. Notably, the proportion of those with OPC under the age of 60 is higher than those with other sub-types of HNC, and the degree to which those with OPC differ to other HNC sub-types with respect to HPV16 E6 positivity is substantial. Table 1 shows that the demographics of those who were selected to have DNA methylation profiled were sufficiently representative of others with the OPC sub-type in HN5000 with respect to exposure to prognostic factors, albeit not necessarily representative of HNC as a whole.

**Table 1:** Comparison of patient demographics in OPC samples selected for methylation data extraction; all samples in HN5000 identified as OPC and all samples in HN5000

Variable	OPC in HN5000 with methylation data and complete phenotype data (*N* = 409)	OPC in HN5000 (*N* = 1909)	All HN5000 (all sub-types) (*N* = 5392)
ICD group (% oropharynx)	100	100	35.4
Sex (% female)	27.0	21.9	27.2
Age (% < 60)	58.4	52.4	42.7
Smoking (% never smoked)	27.1	28.0	24.6
Alcohol (% non-drinker)	25.9	26.6	28.4
HPV16 E6 (% negative)	33.3	32.3	72.0
Survival (% died, prior to 30/09/2017)	26.2	24.2	28.0

### Smoking single-site EWAS and DMR associations

**Fig. 1:**
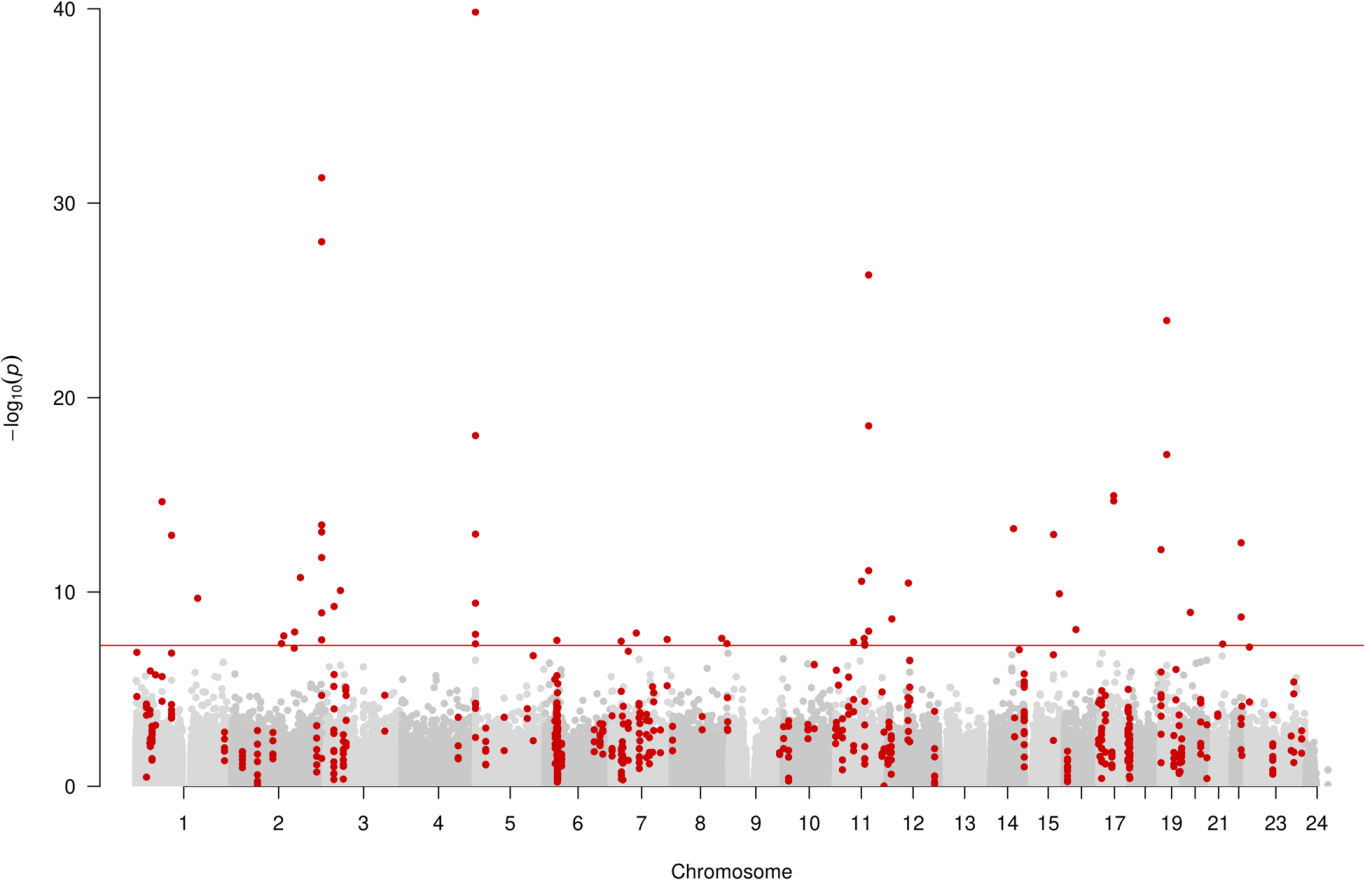
Manhattan plot of EWAS of ever vs never smoking, showing CpG sites within DMRs in red. Each dot represents the EWAS result for a single CpG site, plotting – log_10_ (*P*) (*y*-axis) against the genomic position of the CpG site (*x*-axis). The horizontal red line is at *P* < 5.7 × 10^*−*8^ and represents the value below which CpG sites were considered to have good evidence of association with smoking

Our single-site EWAS of ever vs never smokers (303 ever smokers vs 106 never smokers) revealed 52 CpG site associations annotated to 27 unique loci (*P* < 5.7 × 10^−8^, Bonferroni adjusted *P* < 0.05 for 862,491 tests) (Fig. 1). The CpG site cg05575921, which annotates to the *AHRR* gene region, was most strongly associated (*P* < 1.48 × 10^−40^) and also showed the largest effect size of − 29.5% difference (95% CI − 26.9 to − 32.1%) to in methylation between ever and never smokers. Forty-nine CpG sites had lower DNA methylation in ever smokers, with a mean difference in methylation of − 8.3% (SD, 5.1%; range, − 29.5 to − 2.2%). The three remaining CpG sites had higher methylation in smokers, with a mean difference of 7.7% (SD, 4.2%; range, 4.7 to 12.6%). Supplementary Table 1 provides the complete list of all CpGs that were differentially methylated below a less stringent threshold of *P* = 2.4 × 10^−7^. Of the results presented in this table, 37.5% (24/64 CpGs) were present on the EPIC array but not its 450K predecessor (which provided measurements for 485,512 CpG sites; 93% of these are measured by the EPIC array).

In the differentially methylated region (DMR) analysis (see the “Methods” section) of ever vs never smoking, 166 unique DMRs containing 617 measured CpGs and annotating to 156 gene regions were discovered (Fig. 3). The DMR with the strongest association consisted of 3 measured CpGs (cg21566642, cg01072057 and cg13903162) and was located at Chr2:233284661-233285290, an intergenic CpG island on 2q37.1 (*P* 1.13 × 10^−46^).

### Alcohol consumption single-site EWAS and DMR associations

**Fig. 2:**
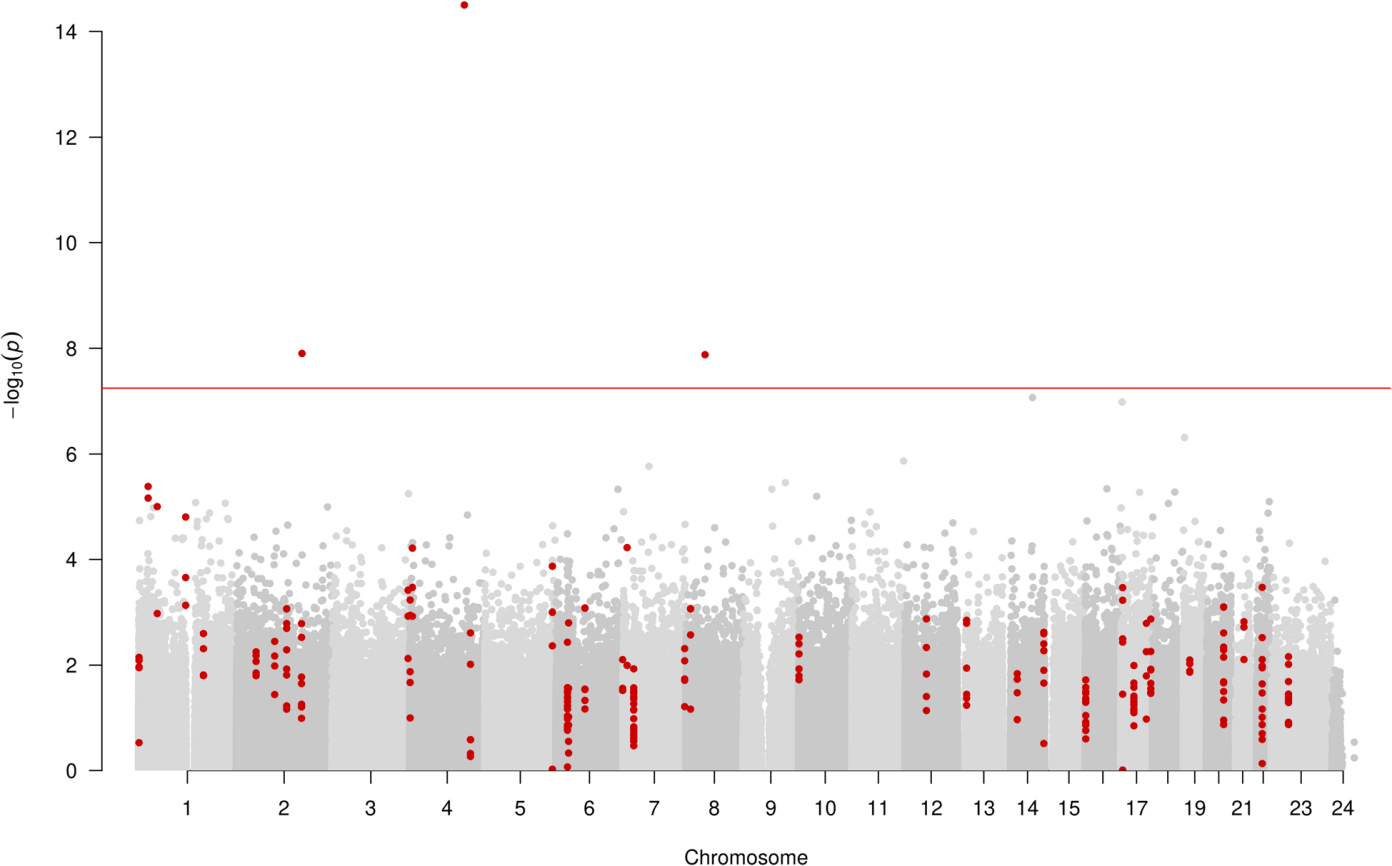
Manhattan plot of EWAS of alcohol consumption, showing CpG sites within DMRs in red. Each dot represents the EWAS result for a single CpG site, plotting – log_10_ (*P*) (*y*-axis) against the genomic position of the CpG site (*x*-axis). The horizontal red line is at *P* < 5.7 × 10^*−*8^ and represents the value below which CpG sites were considered to have good evidence of association with alcohol consumption

The EWAS of alcohol consumption (median 22.5 units/week in 303 alcohol drinkers and 106 non-drinkers) revealed 3 CpG site associations annotated to 3 unique genes (*P* < 5.7 × 10^−8^) (Fig. 2). The association with the smallest *P* value was cg06690548 (*P* 8.3 × 10^−16^), annotating to the *SLC7A11* gene region. This CpG site also showed the largest effect size of − 0.10% difference in methylation per unit increase in alcohol. All results below a multiple testing threshold of 2.4 × 10^−7^ are shown in Supplementary Table 2. Of the results presented in this table, 40% (2/5 CpGs) were present on the EPIC array but not the 450K predecessor.

In the DMR analysis of alcohol consumption, 40 unique DMRs containing 238 measured CpGs and annotating to 34 gene regions were identified (Fig. 2). The DMR with the smallest *P* value was a region of 2 CpGs (cg06690548 and cg13903162) found at Chr4:139162808-139163020 (*P*, 1.45 × 10−^10^), annotated to the *SLC7A11* gene region.

### HPV16 E6 serology single-site EWAS and DMR associations

**Fig. 3:**
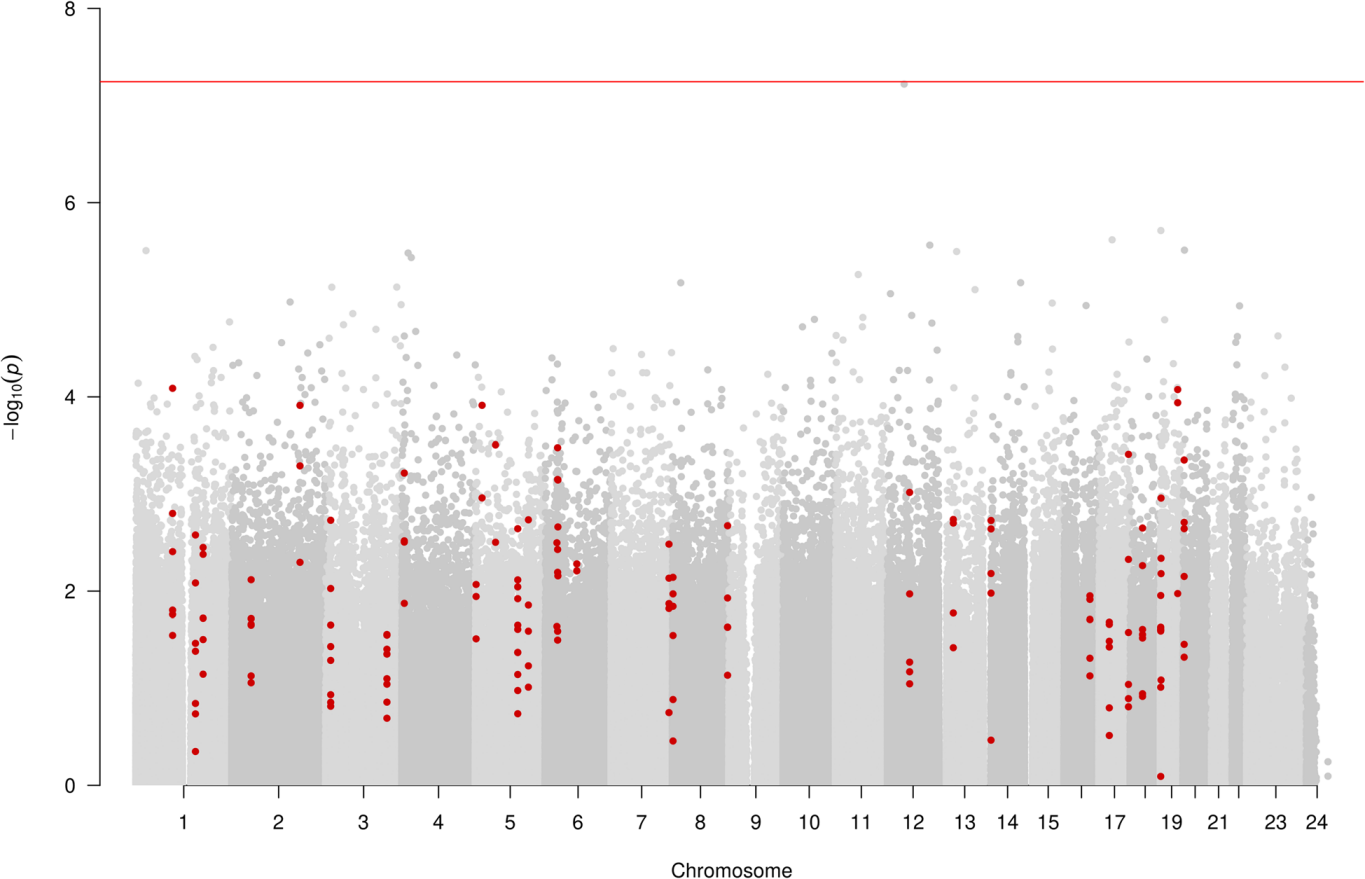
Manhattan plot of EWAS of HPV16E6 seropositivity, showing CpG sites within DMRs in red. Each dot represents the EWAS result for a single CpG site, plotting – log_10_ (*P*) (*y*-axis) against the genomic position of the CpG site (*x*-axis). The horizontal red line is at *P* < 5.7 × 10^−8^ and represents the value below which CpG sites were considered to have good evidence of association with HPV16 E6 seropositivity

In the EWAS analysis of HPVE6 seropositivity (272 seropositive for HPV16 E6 vs 136 seronegative for HPV16 E6), no CpGs passed the *P* value threshold after Bonferroni correction for 862,491 tests (*P* < 5.7 × 10^−8^) (Fig. 3). At a suggestive threshold of 2.4 × 10^−7^, only 1 CpG site (cg26738437; *P*, 1.3 × 10^−7^) was found, annotating to the *CCL16* gene. This probe is not found on the 450K array. Methylation was on average 2.3% lower in HPV16 E6 seropositive participants than controls.

In the DMR analysis of HPV16 E6 seropositivity, 31 unique DMRs pertaining to 158 CpGs and annotating to 38 gene regions were identified (Fig. 3). The most associated DMR was a region of 13 CpGs found at Chr5:110062343-110062838 (*P*, 4.10 × 10−^6^), annotating to the *TMEM232* gene region.

### Survival (~ 3-year) single-site EWAS and DMR associations

**Fig. 4:**
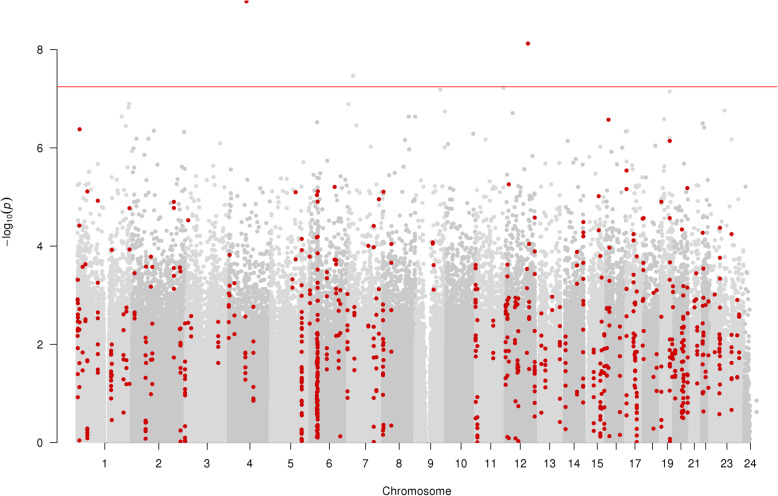
Manhattan plot of EWAS of survival (model 1, not adjusted for smoking, alcohol consumption and HPV16E6 seropositivity), showing CpG sites within DMRs in red. Each dot represents the EWAS result for a single CpG site, plotting – log_10_ (*P*) (*y*-axis) against the genomic position of the CpG site (*x*-axis). The horizontal red line is at *P* < 5.7 × 10^−8^ and represents the value below which CpG sites were considered to have good evidence of association with survival

Of the participants with OPC who had methylation data available, 26.2% had died at the time of censoring (~ 3 years post-diagnosis). In the single-site analysis of survival (model 1, adjusting for age, sex and surrogate variables obtained by SVA [31]), 3 CpGs annotated to 3 unique loci showed association with survival below a Bonferroni threshold for 862,491 tests (*P* < 5.7 × 10^−8^) (Fig. 4). One of the 3 CpGs passing our multiple testing correction showed lower methylation in those who died, while the other 2 CpG sites passing multiple testing correction showed higher methylation in those who died. The site showing lower methylation was also the most strongly associated with survival, annotating to *PAQR3* and showing the largest effect size among our top hits (cg25864218; β [difference in methylation between those who died before 30th September 2017]: − 2.54%; *P* 1.04 × 10^−9^). Of the 2 sites showing higher methylation in those who died, the mean difference in methylation was 0.3% (SD, 0.27%; range, 0.11 to 0.49%). These sites annotated to *DNAH11* (cg07377396; β, 0.49%; *P*, 3.39e−8) and *MYBPC1* (cg12151015; β, 0.11%; *P*, 7.51 × 10^−9^). All results below a suggestive multiple testing threshold of 2.4 × 10^−7^ are shown in Supplementary Table 3. Of the results presented in this table, 47% (7/15) were novel associations, pertaining to the EPIC array vs the 450K predecessor. A heatmap showing the correlation between all CpG sites below the suggestive multiple testing threshold across alcohol consumption, HPV16E6 seropositivity, smoking and survival (model 1) EWAS can be seen in Supplementary Figure 1.

In the DMR analysis of survival (model 1), 142 unique DMRs pertaining to 805 CpGs and annotating to 153 gene regions were identified (Fig. 4). The DMR with the lowest *P* value was a region of 10 CpGs found at Chr17:33814297-33814897 (*P*, 5.26 × 10^−21^), annotating to the *CDK16* gene region.

In our post-hoc sensitivity analyses, we found SVs significantly correlated (Pearson’s product-moment coefficient *P* value < 0.05) with treatment type, TNM stage and neutrophil-to-lymphocyte ratio, a marker of immune profile. Six SVs were associated with laser surgery (Supplementary Figure 2), 3 with surgical removal of an OPC primary (Supplementary Figure 3), 2 with neck resection surgery (Supplementary Figure 4), 6 with teletherapy (Supplementary Figure 5), 4 with chemotherapy (Supplementary Figure 6), 4 with chemoradiotherapy (Supplementary Figure 7), 9 with TNM stage (Supplementary Figure 8) and 4 with neutrophil-to-lymphocyte ratio (Supplementary Figure 9). Furthermore, despite the blood being taken prior to treatment, all 63 SVs explained 29.5% of the phenotypic variance for laser surgery, 15.5% for surgical removal of an OPC primary, 15.0% for neck resection surgery, 20.9% for teletherapy, 21.2% for chemotherapy, 22.3% for chemoradiotherapy, 27.8% for TNM stage and 51.4% for neutrophil-to-lymphocyte ratio.

**Fig. 5:**
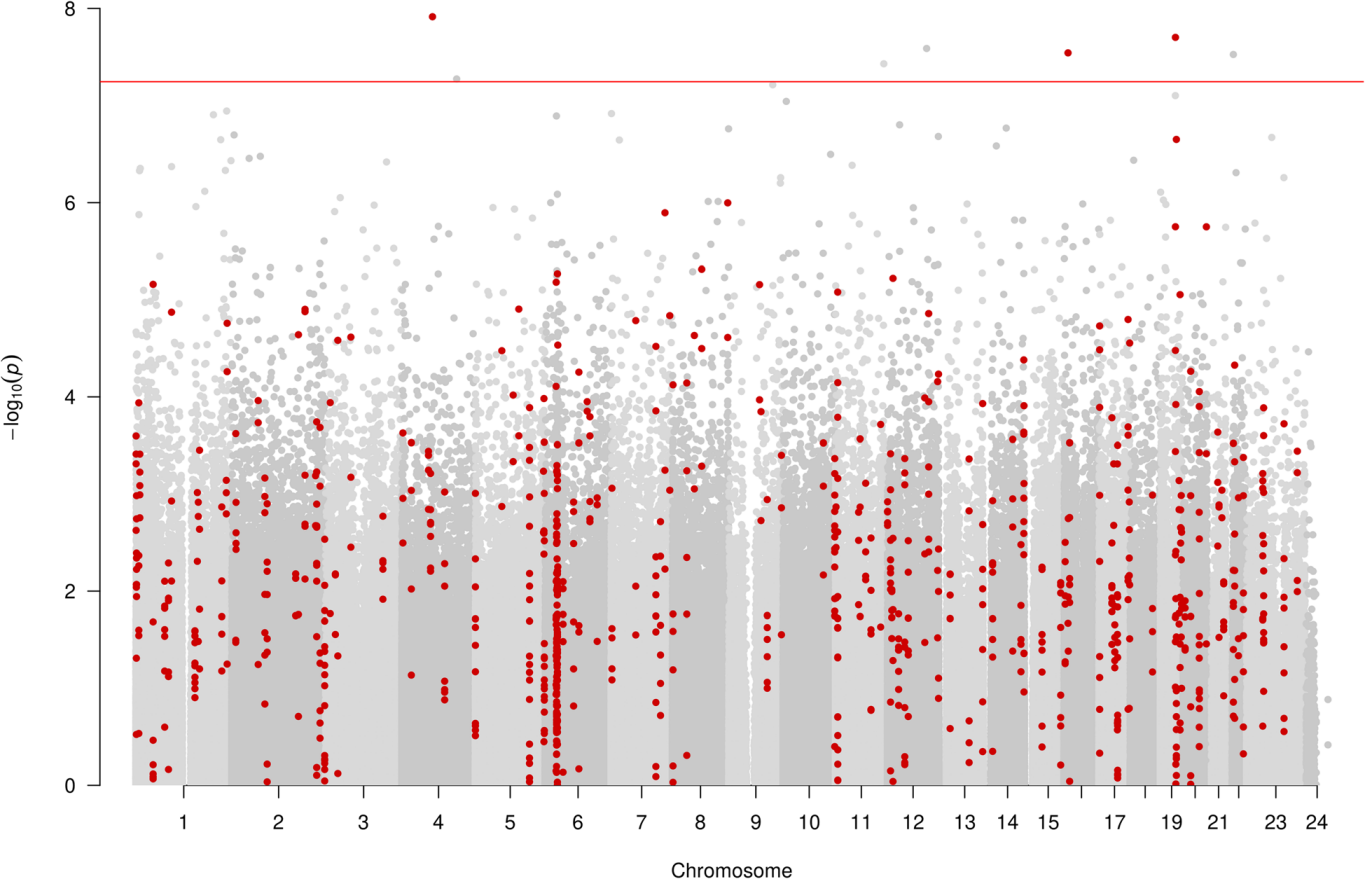
Manhattan plot of EWAS of survival (model 2, adjusted for smoking, alcohol consumption and HPV16E6 seropositivity), showing CpG sites within DMRs in red. Each dot represents the EWAS result for a single CpG site, plotting – log_10_ (*P*) (*y*-axis) against the genomic position of the CpG site (*x*-axis). The horizontal red line is at *P* < 5.7 × 10^−8^ and represents the value below which CpG sites were considered to have good evidence of association with survival

In the single-site analysis of survival with additional adjustment for HPV16E6 seropositivity, smoking status and alcohol intake, 6 CpGs annotated to 4 unique loci showed a *P* value of association below the Bonferroni threshold (*P* < 5.7 × 10^−8^) (Fig. 5). Our most associated site (*P*, 1.22 × 10^−8^), cg25864218, annotates to the *PAQR3* gene region. This site also showed the largest effect size of a − 2.5% difference in methylation between those who died and those who survived. Two CpG sites, cg25864218 (annotating to *PAQR3*, above) and cg12151015 (annotating to *MYBPC1*), showed an association with survival across both adjusted and unadjusted analyses. Other CpGs passing our multiple testing correction which were annotated to genes included *MYBPC1* (cg12151015; β, 0.11%; *P*, 2.59 × 10^−8^), *GRIN2A* (cg08204867; β, − 0.16%; *P*, 2.87 × 10^−8^) and *IL15* (cg26269613; β, 0.67%; *P*, 5.34 × 10^−8^). All results below a suggestive multiple testing threshold of 2.4 × 10^−7^ are shown in Supplementary Table 4. Interestingly, of the results presented in this table, all 23 were novel associations, pertaining to the EPIC array vs the 450K predecessor.

In the DMR analysis of survival (model 2), 157 unique DMRs pertaining to 874 CpGs and annotating to 177 gene regions were identified (Fig. 5). The DMR with the lowest *P* value was a region of 12 CpGs found at ChrX: 47077168-47077877 (*P*, 1.08 × 10^−21^), annotating to the *CDK16* gene region.

### Overlap between risk factor and survival DMRs

**Fig. 6:**
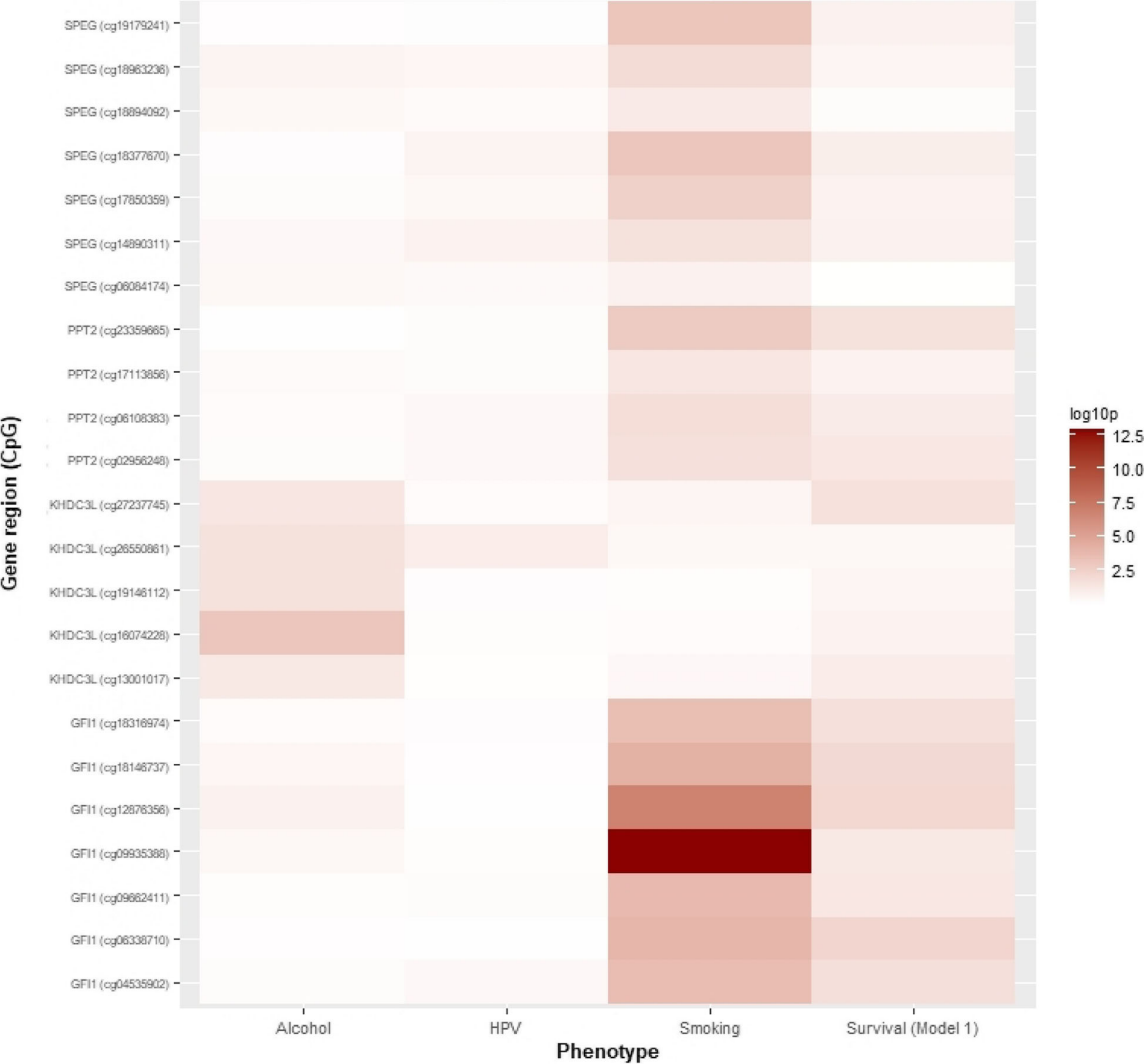
Heatmap showing correlation between differentially-methylated regions (Sidak < 0.05) for each prognostic factor (alcohol consumption, HPV16 E6 seropositivity and smoking) and survival EWAS (model 1 ~ 3-year survival adjusted for age sex and surrogate variables). Strength of association is shown by depth of colour; deeper red denotes a stronger phenotypic association with DMR

Eighteen unique CpGs overlapped between all smoking DMRs and survival (EWAS model 1) DMRs, belonging to 3 unique DMRs (annotated to *GFI1*, *SPEG* and *PPT2*); five CpGs overlapped between all alcohol DMRs and survival (EWAS model 1) DMRs, all pertaining to a single DMR (annotated to *KHDC3L*) (Supplementary Table 5). No CpGs overlapped between the HPV DMRs and survival (EWAS model 1) DMRs. Strength of correlation between CpGs within the overlapping DMRs can be seen in Fig. 6.

Of the 18 CpGs which overlapped between smoking and survival, 15 possessed mQTL proxies in summary data of the genetic determinants influencing methylation levels in 5101 individuals from the Generation Scotland cohort. Of the 5 CpGs which overlapped between alcohol and survival, 3 possessed mQTL proxies in the Generation Scotland summary data (Supplementary Table 5).

### Mendelian randomization analysis of the effect of DNA methylation on OPC survival

**Fig. 7:**
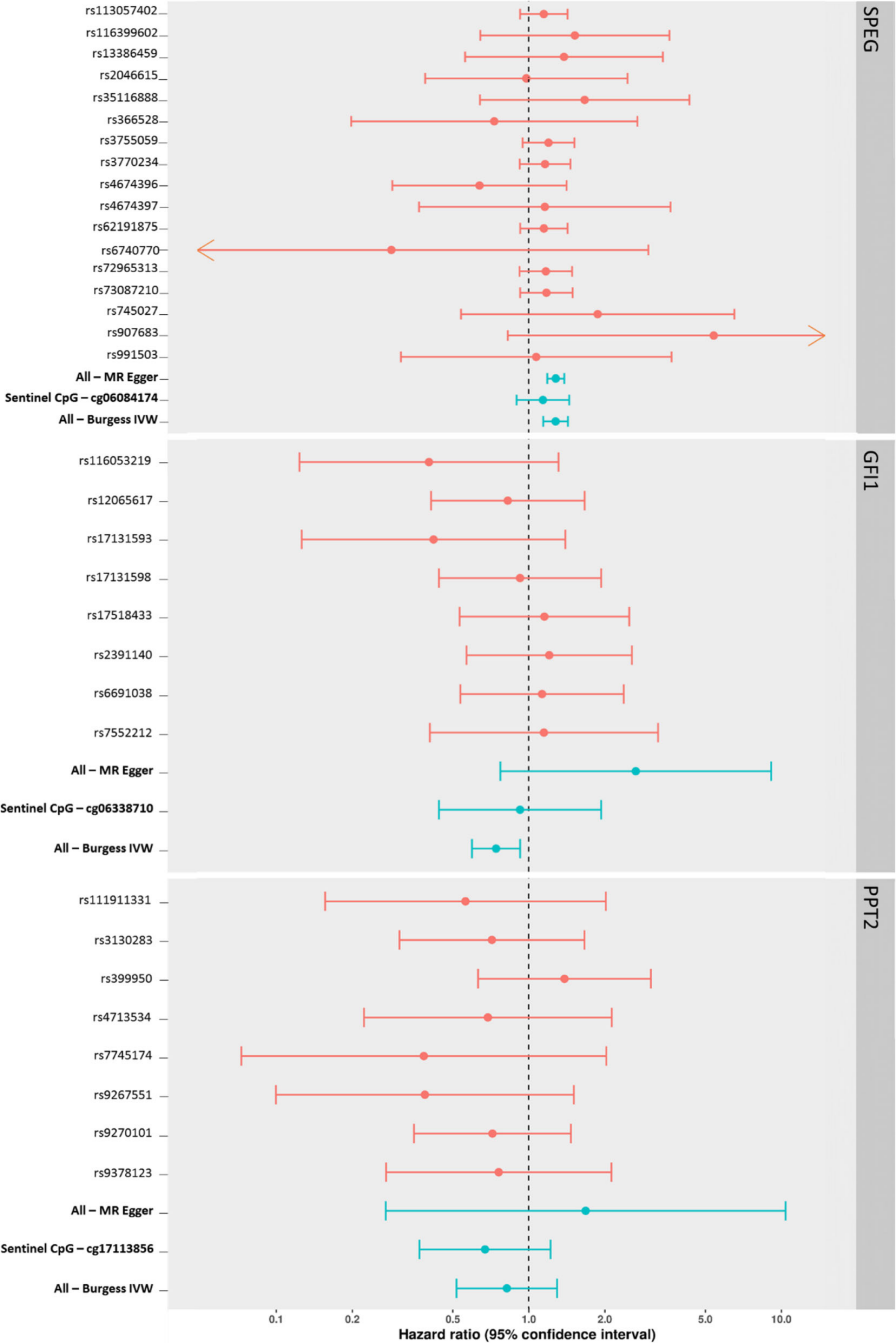
Forest plots showing SNP-specific and overall IV hazard ratio estimates (95% CI) for Mendelian randomization analyses of smoking-associated methylation at 3 gene loci (GFI1, PPT2, SPEG), against 3-year survival in oropharyngeal cancer

Table 2 and Fig. 7 show the results of the MR analysis of DNA methylation on 3-year survival in HN5000, using mQTL-proxied DNA methylation at CpG sites associated with both smoking and survival. Results indicate a causal effect of decreased DNA methylation on survival at the *SPEG* gene locus (Table 2; Chr2:22035443-22036041; HR, 1.28; 95% CI, 1.14 to 1.43), suggesting that DNA methylation may mediate part of the association seen between smoking and increased survival at this gene region. The *GFI1* and *PPT2* (Table 2) gene regions appear to show no consistent evidence of a causal effect of DNA methylation on survival.

**Table 2:** Mendelian randomization (MR) analysis results, assessing epigenetic mediation between smoking status and ~ 3-year survival at the SPEG gene (chromosome 2:220325443-220326041), GFI1 gene (chromosome 1:92946132-92947588) and PPT2 gene (chromosome 6:32120895-32120907). Inverse-variance weighted (IVW) and MR Egger results, adjusted for genetic correlation between mQTLs, are reported as hazard ratios with 95% confidence intervals

Region (gene)	MR method	SNPs	HR	95% CI	***P***
**All DMR CpGs**					
Chr2:220325443-220326041 (*SPEG*)	IVW	17	1.28	1.14 to 1.43	2.12 × 10^−05^
Chr2:220325443-220326041 (*SPEG*)	MR Egger	17	1.28	1.18 to 1.38	4.04 × 10^−10^
Chr1:92946132-92947588 (*GFI1*)	IVW	8	0.74	0.60 to 0.93	7.9 × 10^−03^
Chr1:92946132-92947588 (*GFI1*)	MR Egger	8	2.65	0.77 to 9.12	0.12
Chr6:32120895-32120907 (*PPT2*)	IVW	8	0.82	0.52 to 1.30	0.40
Chr6:32120895-32120907 (*PPT2*)	MR Egger	8	1.68	0.27 to 10.38	0.58
**Sentinel CpG only**					
cg06084174 (*SPEG*)	IVW	3	1.14	0.90 to 1.45	0.29
cg06338710 (*GFI1*)	Wald ratio	1	0.93	0.47 to 1.85	0.84
cg17113856 (*PPT2*)	IVW	2	0.67	0.37 to 1.22	0.19

**Fig. 8:**
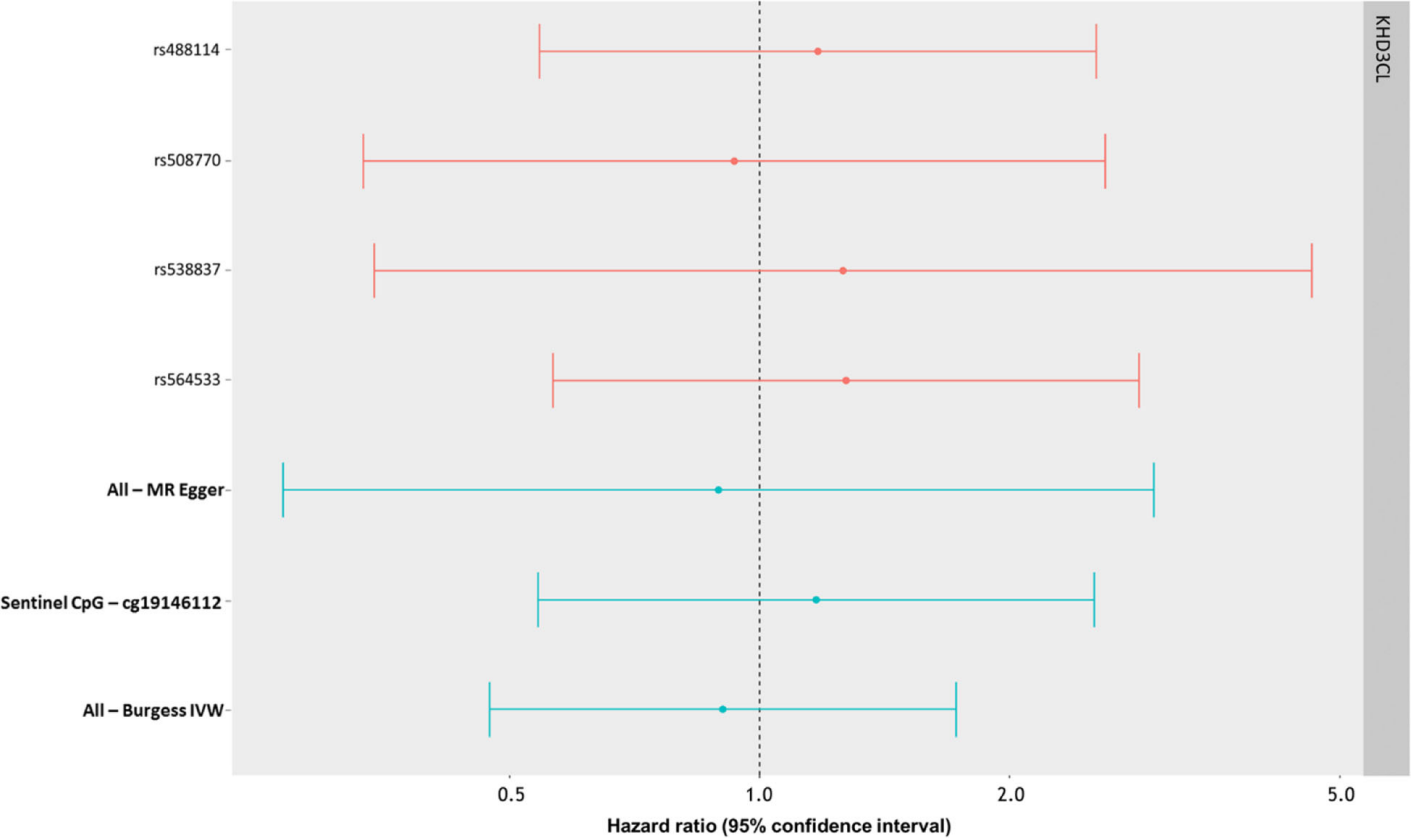
Forest plot showing the SNP-specific and overall IV hazard ratio estimates (95% CI) for Mendelian randomization analyses of alcohol-associated methylation at the KHDC3L gene locus, against 3-year survival in oropharyngeal cancer

Table 3 and Fig. 8 show the results of the MR analysis of DNA methylation on 3-year survival in HN5000, using mQTL-proxied DNA methylation at CpG sites associated with alcohol intake and survival. In our analysis, there appears to be no consistent evidence for a causal effect of DNA methylation on survival at the *KHDC3L* gene locus (Chr6:74072255-74072376).

**Table 3:** Mendelian randomization (MR) analysis results, assessing epigenetic mediation between alcohol consumption and ~ 3-year survival at the KHDC3L gene (chromosome 6:74072255-74072376). Inverse-variance weighted adjusted for genetic correlation between mQTLs (IVW), MR Egger and Wald ratio results are each reported as hazard ratios with 95% confidence intervals

Region (gene)	MR method	SNPs	HR	95% CI	***P***
**All DMR CpGs**					
Chr6:74072255-74072376 (*KHDC3L*)	IVW	4	1.17	0.70 to 1.97	0.55
Chr6:74072255-74072376 (*KHDC3L*)	MR Egger	4	0.89	0.27 to 2.98	0.85
**Sentinel CpG only**					
cg19146112 (*KHDC3L*)	Wald ratio	1	1.17	0.54 to 2.53	0.68

## Discussion

By undertaking single-site EWAS and DMR analyses in whole blood, we identified a number of sites in the genome where DNA methylation may mediate the effect of three prognostic factors (smoking, alcohol and HPV16 positivity) and survival from oropharyngeal cancer. We identified CpG sites and DMRs associated with smoking and alcohol consumption, but none associated with HPV seropositivity. We also identified 6 CpGs associated with survival at 3 years post-diagnosis. Twenty-three CpGs at 4 DMRs were identified in both analyses of risk factor and of survival. MR analysis was conducted to assess whether DNA methylation at the identified sites were causally implicated in relation to OPC survival. We found preliminary evidence to support this mediation pathway between smoking and OPC survival at the *SPEG* gene locus.

In relation to smoke exposure, our results replicate loci previously reported in literature, notably in *AHRR* and *PRSS23* [21, 32]. The effect size seen in our EWAS for cg05575921 (*AHRR*) (29.5%) is markedly stronger compared to the largest published smoking EWAS analysis; Joehanes et al. [21] report 18% lower methylation for between current and never smokers (*P*, 4.60 × 10^−26^). A potential explanation of this finding could be that our analysis was conducted in a case-only setting where smoking is one of the predominant risk factors for HNC, and so smoking intensity is likely to be higher compared to non-cancer smoking populations. We completed a lookup of our top smoking CpG sites (*P* < 5.7 × 10^−8^), using the EWAS Catalog (http://www.ewascatalog.org/) online tool to compare whether our effect sizes were consistently stronger than other published smoking EWAS findings (Supplementary Table 6). Of our 52 sites below a conservative Bonferroni correction, 20 had not been previously reported in published EWAS. The other 32 CpG sites which had previously been reported in literature showed consistently larger effect estimates in response to smoking, in our analysis, when compared to a weighted mean (weighted by sample size) of published EWAS beta values.

Using the same EWAS Catalog resource, we also attempted to determine those associations below our multiple testing threshold for all of our EWAS (Supplementary Table 6). All 5 associations found in our alcohol consumption analysis had not been previously reported in published EWAS of alcohol consumption, likely because they are not measured on the 450k array. *SLC7A11*, the gene annotated to our top CpG site for the alcohol consumption analysis, is essential for glutathione synthesis, a component of the KEAP1-NRF2-CUL3 axis and strongly associated with poor prognosis in The Cancer Genome Atlas (TCGA) HNC cohort [33, 34].

For our 3-year survival EWAS, none of the top associations at *P* < 5.7 × 10^−8^ in either model have previously been reported in published studies. Both survival EWAS models gave a top hit annotating to the *PAQR3* gene (cg25864218). Aberrant promotor methylation at this gene has been shown to be associated with prostate cancer [35], with the gene itself an established tumour suppressor [36]. Within the context of HNC, *PAQR3* has been associated with tumorigenesis in oesophageal cancer [37, 38], though to our knowledge no current literature has examined whether this gene affects oropharyngeal cancer specifically.

In four gene regions, DNA methylation signals were found to overlap between respective prognostic factors and survival: *SPEG* (smoking), *GFI1* (smoking), *PPT2* (smoking) and *KHDC3L* (alcohol consumption). The *SPEG* gene shows specificity to vascular smooth muscle cells—the major cell type in blood vessel walls, in which smoking has been shown to produce abnormal function throughout the human body [39]. Functional annotations show the *SPEG* gene to be essential for cardiac function in particular, with deficiency of this gene reported to result in heart failure [40]. The *GFI1* gene encodes a zinc finger protein which appears to play a role in diverse developmental contexts such as haematopoiesis and oncogenesis by contributing to the control of histone modifications to silence gene promotors [41]. Parmar et al. suggest that smoking-related epigenetic changes at *GFI1* are robustly associated with cardiometabolic risk factors, with lower methylation at *GFI1* CpGs associated with elevated triglyceride levels [42]. The *PPT2* gene encodes a protein which removes thioester-linked fatty acyl groups from various substrates, including S-palmitoyl-CoA [43]. A genetic variant within this gene region appears to be robustly associated with the ratio of forced expiratory volume in the first second (FEV_1_) to forced vital capacity (FEV_1_/FVC, an indicator of airflow obstruction) in a replicated GWAS of over 20,000 individuals from Cohorts for Heart and Aging Research in Genomic Epidemiology (CHARGE) consortium studies [44]. Finally, the *KHDC3L* gene shows specificity to oocytes and is thought to play a role in the global establishment of methylation in these cells. However, the mechanistic pathway through which these changes occur has yet to elucidated, due in part to the novel discovery of *KHDC3L*’s effect on global methylation currently being a novel discovery [45].

The tight confidence intervals and consistent direction of effect between MR Egger and IVW estimates for the *SPEG* locus provide us with greater confidence in a reliable IV and sufficient statistical power to demonstrate preliminary evidence of a causal effect of methylation at this locus on reduced survival. A lookup in the BIOS QTL Browser (https://genenetwork.nl/biosqtlbrowser/) confirms 20 cis-expression quantitative trait methylations (eQTMs) showing evidence of correlation between gene expression and methylation at this locus in whole blood, though further work evaluating tissue-specific expression is required. The role of this gene in cardiac function is of interest since cardiovascular disease is a common comorbidity of people with HNC [46, 47], where the 5-year incidence of non-cancer survival is 13% [48]. Our finding may have clinical relevance for OPC and prognostic studies more broadly if methylation and/or expression of *SPEG* is confirmed to be causally related to with survival. For example, there may be scope to target DNA methylation at this gene region therapeutically if a proportion of the effect of smoking on mortality is mediated through this pathway. However, appropriate validation and replication studies need to be conducted to establish the true effect of smoking-related DNAm at the *SPEG* gene region on mortality. Furthermore, quantification of the proportion of smoking-related mortality risk at this gene region will be crucial in determining whether targeting it is a cost-effective therapeutic target.

To our knowledge, this is the first EWAS study investigating oropharyngeal cancer survival using a Cox proportional-hazards model to investigate DNA methylation in relation to incident survival at ~ 3 years. A key strength of the study relates to the use of the EPIC array which profiles methylation at approximately twice as many CpG sites as its 450k predecessor. Across the EWAS of smoking, alcohol, HPV and both survival models, 39.4% of the CpG sites at *P* < 2.4 × 10^−7^ were specific to the EPIC array. However, proportionally, our results suggest that associations are not enriched with the inclusion of novel enhancer region CpGs from this array. A one-sided Fisher’s exact test for enrichment of EPIC probes vs 450K probes in CpG sites below *P* 2.4 × 10^−7^ confirms this; *P* > 0.99.

The gold standard to identify HPV-driven tumours is through detection of HPV DNA and RNA. A potential limitation of this study is that our EWAS of HPV16 positivity is based on measures of serological response to HPV infection rather than p16 and/or in-situ hybridisation (ISH), which are typically used in clinical practice in the UK [2]. P16 alone will tend to overestimate the number of HPV-driven tumours, as some tumours that are not HPV-driven can still be p16-positive [49]. However, HPV-driven OPC mount an early and marked serological response that has good agreement with tissue markers, indicating an HPV-driven tumour [50]. In HN5000, current information on p16 status relies solely on clinical information rather than being a baseline measurement across all study centres. Although HN5000 are in the process of carrying out p16 on many participants with OPC, this is not currently available, and so there are considerable missing data for this measure. Additionally, very few centres performed confirmatory tests to the serology which was conducted at baseline, such as ISH. There is good agreement between serology and tissue measures of HPV-driven tumours; studies have found little difference in the number of individuals confirmed as having HPV-driven OPC between serology and p16 [50], with p16 alone more prone to overestimate the number of HPV-driven cases. Given a current lack of p16/ISH data in HN5000, we remain confident in our method of viral diagnosis of OPC.

Most HPV-driven OPC are caused by the HPV16 serotype, to the extent that presence of antibodies specific to HPV16 E6 > 1000 MFI are widely accepted as a reliable measure of “HPV-driven” OPC [51]. However, an estimated 3% of HPV-driven OPC are caused by other HPV sub-types. We were careful to define the HPV EWAS as an EWAS of ‘HPV16 E6 seropositivity’ to reflect the HPV detection method and sub-type specificity. An epigenome-wide investigation in relation to a broader phenotype of HPV seropositivity, including other HPV sub-types, may have identified other CpG sites which were not identified in relation to E6, although this is unlikely given the particularly low proportion of HPV-driven OPC not caused by HPV16.

Collider bias may influence associations between our prognostic factors and survival in a case-only setting [52, 53]. HPV, smoking and alcohol are all associated with OPC incidence. By only examining OPC cases, incidence is conditioned on, potentially inducing an association between HPV, smoking, alcohol and any unmeasured confounding (including, but not limited to, any possible factor independently associated with OPC incidence). By inducing artificial associations between risk factors and confounding in this way, conducting an EWAS stratified by cases may generate spurious associations between methylation changes which do not affect survival, and survival.

Some of our MR analyses highlight potential violations of its methodological assumptions. Primarily, those analyses where the MR Egger estimate shows an effect in the opposite direction to the IVW estimate (*GFI1*, *PPT2*, *KHDC3L*) could indicate an IV where one or more of the genetic variants proxying methylation is biasing the effect due to horizontal pleiotropy. However, for each of these analyses, the MR Egger intercept test of heterogeneity (explained elsewhere [54, 55]) spans 0 (*GFI1* intercept − 0.25, 95% CI −0.54 to 0.05, *P* value 0.10; *PPT2* intercept − 0.18, 95% CI − 0.58 to 0.23, *P* value 0.40; *KHDC3L* intercept 0.07, 95% CI − 0.09 to 0.23, *P* value 0.37), indicating that directional pleiotropy is not causing the difference between the MR Egger and IVW estimates. Consequently, a possible explanation of the opposing directions seen between MR Egger and IVW estimates is that, in this instance, the low power of the MR Egger tests has simply generated imprecise effect estimates.

One notable limitation of our MR analysis is that it is likely particularly conservative; we assessed overlap between prognostic factor DMRs and survival DMRs only if they surpassed our multiple correction threshold in both analyses. We opted for this approach to improve confidence that regional methylation was associated with *both* a prognostic factor and survival. However, in order to reduce the possibility that regional methylation was only associated with a prognostic factor (and only spuriously associated with survival), we may have missed genuine causal mediation at less-stringent *P* value thresholds.

## Conclusion

Within the context of OPC, we found novel epigenetic biomarkers measured by the EPIC array in whole blood to be associated with the prognostic factors of smoking and alcohol, and with survival. Of these biomarkers, we used overlapping signals between prognostic factor and survival analyses to then conduct MR analysis to appraise the causal role of DNA methylation. Using an IVW approach to investigate the causal effect of DNA methylation at the identified sites, we found that a collection of CpGs located within a DMR associated with smoking (located at Chr2:220325443-220326041; annotating to the *SPEG* gene) showed some evidence of a causal effect on decreased survival (HR, 1.28; 95% CI, 1.14 to 1.43; *P*, 2.12 × 10^−05^). DNA methylation at this locus could potentially mediate some of the association between smoking and OPC survival. To strengthen the validity of these findings, replication analyses and a longer follow-up period in Head and Neck 5000 are recommended.

## Methods

### Study population

The study population for this analysis was individuals enrolled in the Head and Neck 5000 (HN5000) clinical cohort study. Participants for our study were selected from the wider pool of individuals in HN5000 (*N*, 5392) based on an ICD-10 coding of oropharynx (CO1, CO5, CO9, C10.0-2, C10.3, C10.8 and C10.9; *N*, 1909/5392), availability of OncoChip genotype data generated previously (*N*, 1034/1909; necessary to conduct the Mendelian randomization analysis—see the “mQTL associations with survival” subsection below) [56], baseline questionnaire and data capture information (see below) and the availability of blood samples taken at baseline (*N*, 448/1034).

Full details of the study methods and overall population are described in detail elsewhere [57, 58]. Briefly, between April 2011 and December 2014, 5511 individuals with HNC were recruited from 76 centres across the UK. All people with a new diagnosis of HNC were eligible to join the study and were recruited before or within a month of their cancer treatment commencing. Individuals with cancers of the pharynx, mouth, larynx, salivary glands and thyroid were included, while those with lymphoma, tumours of the skin or a recurrence of a previous head and neck cancer were excluded from the study.

Local research nurses obtained informed consent from individuals, which included agreement to: collect, store and use biological samples; obtain samples of stored tissue; carry out genetic analyses and collect clinical information from hospital notes and survival data through record linkage. Ethics approval for this study was granted by the National Research Ethics Committee (South West Frenchay Ethics Committee, reference 10/H0107/57, 5th November 2010) and approved by the research and development departments from participating NHS Trusts.

### Baseline data collection

Participants were asked to complete a series of three self-administered questionnaires at recruitment, enquiring about: (1) social and economic circumstances, overall health and lifestyle behaviours; (2) physical and psychological health, well-being and quality of life and (3) past sexual history and behaviours [57]. Information on diagnosis, treatment and co-morbidity was recorded on a short data capture form using questions based on a national audit [51, 59]. Diagnoses were coded using the International Classification of Diseases (ICD) version 10 [60, 61], and clinical staging of the tumour was derived based on the American Head and Neck Society TNM staging [52, 62].

Research nurses collected a blood sample from all consenting participants at recruitment, prior to treatment, unless treatment was their diagnostic procedure [58]. These were then sent to the study centre laboratory at ambient temperature for processing. The blood samples were centrifuged at 3500 rpm for 10 min and the buffy coat layer used for DNA extraction. Any additional samples from the same participant were frozen and stored at – 80 °C.

#### Assessment of tobacco, alcohol and HPV infection

Detailed information on tobacco and alcohol history was obtained at baseline via the self-administered questionnaire. Participants were asked about their current smoking and drinking status and their use of tobacco and alcohol products prior to receiving their HNC diagnosis.

Among smokers, information on age at smoking initiation and number of years of smoking was obtained. The questionnaire differentiated between use of cigarettes, hand-rolled cigarettes, cigars and smokeless tobacco, whereby a cigar was considered equivalent to four cigarettes. From this information, participants were dichotomised into ever and never smokers. Ever smokers were defined as those who smoked at the equivalent of at least 1 tobacco product a day per year or ≥ 100 cigarettes in their lifetime. Never smokers were those who reported not smoking in any of the questions answered.

Respondents were asked to report their average weekly alcohol consumption of a range of beverage types (wine, spirits and beer/larger/cider) before they were diagnosed with cancer. From these measures, we derived an average intake of alcohol consumption in units per week.

HPV serologic testing (HPV16 E6, E7, E1, E2, E4 and L1) was conducted at the German Cancer Research Center (DKFZ, Heidelberg, Germany) using glutathione S-transferase multiplex [63]. Median fluorescence intensity (MFI) values were dichotomized to indicate HPV16 E6 seropositivity using a cut-off of ≥ 1000 MFI [64]. E6 seropositivity is known to be a marker of current infection and has a high sensitivity and specificity for HPV16-driven oropharyngeal cancer [65].

#### Study follow-up and survival

Regular updates were received from the NHS Central Register (NHSCR) and the NHS Information Centre (NHSIC) notifying on subsequent cancer registrations and survival among cohort members in the Head and Neck 5000 study. Recruitment for the study finished in December 2014 and follow-up information on survival status was obtained on 30th September 2017, resulting in at least 2.75 years of follow-up for all participants.

### DNA methylation

#### Data generation

Following extraction, DNA was bisulphite-converted using the Zymo EZ DNA Methylation^TM^ kit (Zymo, Irvine, CA, USA). Genome-wide methylation data were generated using the Infinium MethylationEPIC BeadChips (EPIC array) (Illumina, USA) according to the manufacturer protocol. The arrays were scanned using an Illumina iScan (version 2.3).

#### Pre-processing

Raw data files (IDAT files) were pre-processed using the R package *meffil* (https://github.com/perishky/meffil/) [56]. We used the same R package to perform quality control and normalisation [66]. Sample mismatches and outliers were identified and removed based on allosome methylation (*N*, 2 incorrect sex prediction; *N*, 3 outliers) and 65 genotype probes, which were compared with SNP-chip data from the same individual (*N*, 3 exclusions). Sample outliers were also identified based on control probe (bisulfite 1 and bisulfite 2) mean outliers (*N*, 2 exclusions), outliers for median intensity methylated vs unmethylated signal for all control probes (*N*, 2 exclusions), detection *P* value (*N*, 2 exclusions based on high proportion of undetected probes [> 10% of probes failing a detection *P* value > 0.01]) and low bead numbers (*N*, 1 exclusions). Overall, 443 samples passed QC. Following QC, functional normalization was used to separate biological variation from technical variation [57, 67]. Data were normalised using 5 control probe principal components derived from the technical probes. The Infinium EPIC array pipeline detects the proportion of molecules methylated at each CpG site on the array. For the samples, the methylation level at each CpG site was calculated as a beta value (*β*), which is the ratio of the methylated probe intensity and the overall intensity and ranges from 0 (no cytosine methylation) to 1 (complete cytosine methylation).

### EWAS

Epigenome wide association study (EWAS) analysis was conducted to identify associations between DNA methylation and (1) alcohol consumption, (2) smoking status and (3) HPV16E6 seropositivity. EWAS were conducted in *meffil*, using a linear regression model of DNA methylation regressed on the prognostic factors, adjusting for age, sex, surrogate variables obtained by SVA [31] and the other prognostic factors (e.g. for alcohol intake, adjusting for smoking and HPV16E6). Of the 443 individuals who passed QC, the number of individuals with complete phenotype data for alcohol intake, smoking status and HPV16E6 seropositivity with which to conduct an EWAS was 409 as of the 2018, version 2.3 release of HN5000 data. All of these samples possessed information on survival status.

An EWAS for survival from recruitment (last participant recruited December 2014)—September 2017 (or time of censoring; whichever occurred first)—was conducted using code adapted from the *meffil* R package [56, 66]. Cox proportional-hazards models were employed: model 1 adjusting for age, sex and surrogate variables obtained by SVA [31]; model 2 adjusting for age, sex, surrogate variables obtained by SVA [31], HPV16E6 seropositivity, smoking status and alcohol intake. Death from any cause was used as the failure variable and time to death (or censoring) in days as the time variable.

Due to the large number of tests conducted in our EWAS, we employed a Bonferroni correction to derive a conservative *P* value threshold of 5.7 × 10^−8^ (0.05/862491 independent tests) to determine those sites showing strong evidence of association with our risk factor of interest or survival, respectively. We also used the alpha value calculated for the Illumina 450K array (the predecessor to the MethylationEPIC array) as a *P* value threshold of 2.4 × 10^−7^ for suggestive evidence of association [68].

### DMR analysis

Adjacent probes on the Illumina arrays are often highly correlated; therefore, differentially methylated regions (DMRs) may reveal regions of DNA where CpGs are associated with risk factors and survival. Following each EWAS, we conducted DMR analysis using the *dmrff* R package [59, 69]. This analysis identified regions (> 1 CpG site per region) enriched for low *P* values (*P* < 0.05), corrected for dependencies between other CpG sites in the DMR and adjusted for multiple testing.

### Sensitivity analysis

To ensure our SV analysis was adequately adjusting for factors which influenced our survival EWAS, we systematically assessed the correlation of SVs with treatment type, TNM stage and immune profile post-hoc. Additionally, we appraised the amount of phenotypic variance the SVs explained in the above factors which were available in HN5000 (laser surgery, surgical removal of an OPC primary, neck resection surgery, teletherapy, chemotherapy, chemoradiotherapy, TNM stage and neutrophil-to-lymphocyte ratio).

### Generation Scotland methylation quantitative trait loci

DNA methylation can be influenced by genetic sequence variations, such that individual genotypes at a given locus may result in different patterns of DNA methylation due to allele-specific methylation [70–72]. Such sites, called methylation quantitative trait loci (mQTLs), can influence the methylation pattern across an extended genomic region [61] and can be used as a proxy for methylation levels in a Mendelian randomization (MR) framework. Such sites, called methylation quantitative trait loci (mQTLs), can influence the methylation pattern across an extended genomic region [70] and can be used as a proxy for methylation levels in a Mendelian randomization (MR) framework [29].

To generate mQTLs, methylation data from a quality-controlled subset of individuals (*N*, 5101) from the Generation Scotland: Scottish Family Health Study who had undergone EPIC array DNA methylation profiling, described previously [73], were used. Following measurement of DNA methylation, normalization was performed using the R package *minfi* [65, 74], producing *M* values [66, 75] for downstream analysis. Briefly, linear mixed modelling was used to remove potential effects from technical factors, adjusting for both fixed and random effects. Fixed effects included the top 50 principal components of control probe intensities (explaining 99% of variation in control probe intensities) [67, 76], clinic centre for blood draw appointment, processing batch, year of clinic visit and sentrix position (position of the sample on EPIC array slide). Random effects included blood draw appointment date and sentrix ID (EPIC array slide). The model converged successfully for 712,595 sites. Outliers from this normalisation with residualized-M-values more than five interquartile ranges from the nearest quartile were removed [77].

A GKFSC model [78, 79] was then fitted to derive mQTLs from the normalised data, including 5 matrices as random effects, and other covariates as fixed-effects. The matrices were G (a genomic relationship matrix), K (a kinship relationship matrix) [80, 81], F (an environmental matrix representing nuclear-family-member relationships), S (an environmental matrix representing full-sibling relationships) and C (an environmental matrix representing couple relationships) [78, 79]. Covariates (as fixed effects) included age, age^2^, gender, estimated cell counts, season of clinic visit, appointment time of the day and appointment day of the week. The model successfully converged in 638,737 CpG sites.

### Generation of instrumental variables for DMRs

Prior to MR analysis being conducted, we generated instrumental variables (IVs) proxying CpG sites identified in analyses of both prognostic factors and survival (Supplementary Figure 1). Where possible, we found DMRs (*P* < 0.05) from our analyses for each prognostic factor and looked for a corresponding DMR in our survival analysis (model 1, unadjusted for prognostic factors). CpG sites common to DMRs found this were retained.

Next, using the summary genetic data for mQTLs from Generation Scotland, we extracted all mQTLs proxying any CpG site per DMR grouping (MAF > 0.05; *P* < 5 × 10^−8^). From this list, we generated instruments by LD pruning iteratively; first taking all mQTLs associated with the sentinel CpG (defined as the CpG in each DMR with the lowest *P* value) and clumping with an *r*^2^ of 0.01. We then took the second most associated CpG in the DMR and extracted all mQTLs associated with it which were not associated with the previous CpG. The remaining mQTLs were then clumped and combined with the mQTLs proxying the sentinel CpG. This process was repeated for each CpG within a DMR. Clumping and mQTL extraction were conducted using R 3.4.1, with the *TwoSampleMR* R package [82].

In order to account for mQTL proxies influencing methylation at multiple CpG sites, we conducted a meta-analysis of mQTL-CpG effects. Per DMR, we used the *metafor* R package [74, 83] to meta-analyse each mQTL effect (beta) on methylation levels at each CpG using a restricted maximum likelihood (REML) model, adjusting for pairwise correlation between the CpG sites proxied by our instrument. From this, we obtained an mQTL effect on average methylation levels across the DMR.

### mQTL associations with survival

The mQTLs identified above were then regressed against survival in HN5000, using the SurvivalGWAS_SV program to run Cox proportional-hazards survival analyses with an additive dosage model for each of the selected SNPs [84]. Death from any cause was used as the failure variable and time to death (or censoring) in days as the time variable. Age at cancer diagnosis and sex were used as covariables in the model. For each SNP, the log-hazard ratio (and standard error) per minor allele was reported.

### Power calculation for Mendelian randomization analyses

**Fig. 9:**
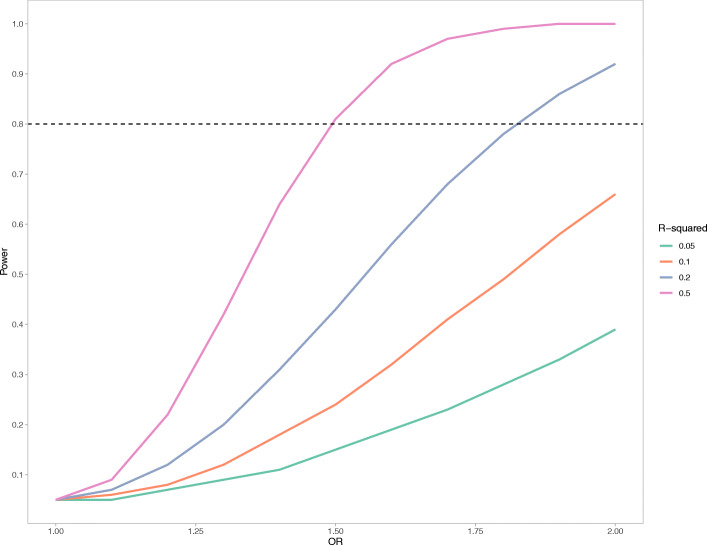
Smoothed-line plot showing minimum expected power for MR analyses using mQTLs to proxy gene regions. This figure denotes a lower bound in expected power at different OR values rather than a definitive estimate. Sample size = 409; alpha = 0.05. Each plot denotes a different proportion of variance explained, ranging from 5 to 50%

Given the use of mQTL summary-level data rather than individual-level data, we could not calculate the exact variance explained of methylation across the multiple DMR CpG sites we proxied. Therefore, we calculated power for our analyses based on largest variance explained in methylation by a single mQTL for each region analysed, in the knowledge that this would constitute a minimum bound of the total variance explained when combining multiple instruments. Accordingly, our power calculations show an extremely conservative, minimum estimate of power to conduct MR analyses. Power was calculated using the mRnd power calculator (http://cnsgenomics.com/shiny/mRnd/) using an alpha of 0.05 and sample size of 409 at different OR values, for a range of *r*^2^ values (Fig. 9).

### Mendelian randomization analyses

Following identification of shared methylation patterns between prognostic factors and OPC survival, we attempted to ascertain whether methylation was a true causal intermediate, or simply just associated with both prognostic factors and survival. To this end, we conducted Mendelian randomization to appraise the causal effect of DNA methylation on survival. To achieve this, we conducted a two-sample MR analysis. In the first sample, we used mQTL-DMR effect estimates (βGP) from Generation Scotland, and in the second sample, mQTL-survival estimates (βGD) from HN5000. For each mQTL, we calculated the log HR per unit (β) increase in DNA methylation at the DMR by the formula βGD/βGP (Wald ratio). Standard errors were approximated by the delta method. Where multiple mQTLs were available for one DMR, these were combined in a fixed effects meta-analysis after weighting each ratio estimate by the inverse variance of their associations with the outcome (IVW approach). In order to account for correlation between mQTLs, we adjusted for genetic correlation using LDMatrix [76, 85] to generate a genetic correlation matrix (1000 Genomes reference standard) of mQTLs, which was included as a covariate in our MR regression analysis [86]. In addition to our main analysis detailed above, we conducted multivariable MR Egger analysis as an assessment of IV heterogeneity. We also conducted sensitivity MR analyses by calculating the log HR per unit increase in DNA methylation for the single most-associated CpG with each DMR we analysed—as above, Wald ratios were calculated for CpGs proxied by a single mQTL, and IVW MR estimates were calculated when multiple mQTLs were available to proxy a CpG.

## Supplementary information

**Additional file 1:Supplementary Figure 1.** Heatmap showing correlation between top CpG sites (P<1x10-7) from each prognostic factor (alcohol consumption, HPV16 E6 seropositivity and smoking) and survival EWAS (Model 1: ~3-year survival adjusted for age sex and surrogate variables; Model 2: as Model 1, additionally adjusted for HPV16E6 seropositivity, smoking status and alcohol intake). Strength of association is shown by depth of colour; deeper red denotes a stronger phenotypic association with a hypermethylated CpG and deeper cyan denotes a stronger phenotypic association with a hypomethylated CpG. **Supplementary Figure 2.** - Surrogate variables correlated at P<0.05 (Pearson's) with laser surgery in HN5000. **Supplementary Figure 3.** - Surrogate variables correlated at P<0.05 (Pearson's) with surgery on an OPC primary tumour in HN5000. **Supplementary Figure 4.** - Surrogate variables correlated at P<0.05 (Pearson's) with neck resection surgery in HN5000. **Supplementary Figure 5.** - Surrogate variables correlated at P<0.05 (Pearson's) with teletherapy in HN5000. **Supplementary Figure 6.** - Surrogate variables correlated at P<0.05 (Pearson's) with chemotherapy in HN5000. **Supplementary Figure 7.** - Surrogate variables correlated at P<0.05 (Pearson's) with chemoradiotherapy in HN5000. **Supplementary Figure 8.** - Surrogate variables correlated at P<0.05 (Pearson's) with TNM stage in HN5000. **Supplementary Figure 9.** - Surrogate variables correlated at P<0.05 (Pearson's) with neutrophil-to-lymphocyte ratio in HN5000.

**Additional file 2: Supplementary Table 1.** Genome-wide differentially-methylated CpG sites associated with smoking status below a multiple testing threshold of P < 2.4e-07. Results are adjusted for age, sex, surrogate variables obtained by SVA, alcohol consumption and HPV16E6 seropositivity. **Supplementary Table 2.** - Genome-wide differentially-methylated CpG sites associated with alcohol consumption below a multiple testing threshold of P < 2.4e-07. Results are adjusted for age, sex, surrogate variables obtained by SVA, smoking status and HPV16E6 seropositivity. **Supplementary Table 3.** - Genome-wide differentially-methylated CpG sites associated with ~3-year survival below a multiple testing threshold of P < 2.4e-07. Results are adjusted for age, sex and surrogate variables obtained by SVA. **Supplementary Table 4.** Genome-wide differentially-methylated CpG sites associated with ~3-year survival below a multiple testing threshold of P < 2.4e-07. Results are adjusted for age, sex, surrogate variables obtained by SVA, smoking status, alcohol consumption and HPV16E6 seropositivity. **Supplementary Table 5.** Genetic instrumental variables (IVs) used in Mendelian randomization analyses to assess epigenetic mediation between prognostic factors and ~3-year survival. The final # SNPs denotes genetic IVs which both proxy a CpG and where the same position is available in the genome-wide association study of 3-year survival survival. **Supplementary Table 6.** Lookup of CpG sites in the MRCIEU EWAS Catalog across all EWAS analyses below a Bonferroni p-value threshold of 5.7e-08. Betas for all studies reporting beta values are calculated as a weighted mean, weighted by sample size. **Supplementary Table 7.** CpG sites (P<2.4e-7) associated with ~3-year survival adjusted for age, sex and surrogate variables obtained by SVA, compared against betas, standard errors and p-values at the same sites when comorbidity and stage are included as additional covariates in the EWAS model.

## Data Availability

The datasets analysed during the current study are available from the Head and Neck 5000 study upon submission of a research proposal. If you would like to access this resource, please contact the Head and Neck 5000 Executive on headandneck5000@uhbristol.nhs.uk. The study website http://www.headandneck5000.org.uk/ describes the resource and the types of data available.

## Abbreviations

CpG: Cytosine-phosphate-guanine
CHARGE: Cohorts for Heart and Aging Research in Genomic Epidemiology
CI: Confidence interval
DNAm: DNA methylation
DMR: Differentially methylated region
EWAS: Epigenome-wide association study
FEV: Forced expiratory volume
FVC: Forced vital capacity
HN5000: Head and Neck 5000
HNC: Head and neck cancer
HPV: Human papillomavirus
HR: Hazard ratio
ICD: International Classification of Diseases
ISH: In-situ hybridisation
IV: Instrumental variable
IVW: Inverse-variance weighted
MAF: Minor allele frequency
MFI: Median fluorescent intensity
mQTL: Methylation quantitative trait loci
MR: Mendelian randomization
NHS: National Health Service
OPC: Oropharyngeal cancer
QC: Quality control
SD: Standard deviation
SVA: Surrogate variable análisis
TGCA: The Cancer Genome Atlas
TNM: Tumour node metastasis
