# Supplementary material for: Identifying epigenetic biomarkers of established prognostic factors and survival in a clinical cohort of individuals with oropharyngeal cancer
Source: Clin Epigenetics. 2020 Jun 29;12:95. doi: 10.1186/s13148-020-00870-0 (PMC7322918; doi:10.1186/s13148-020-00870-0)

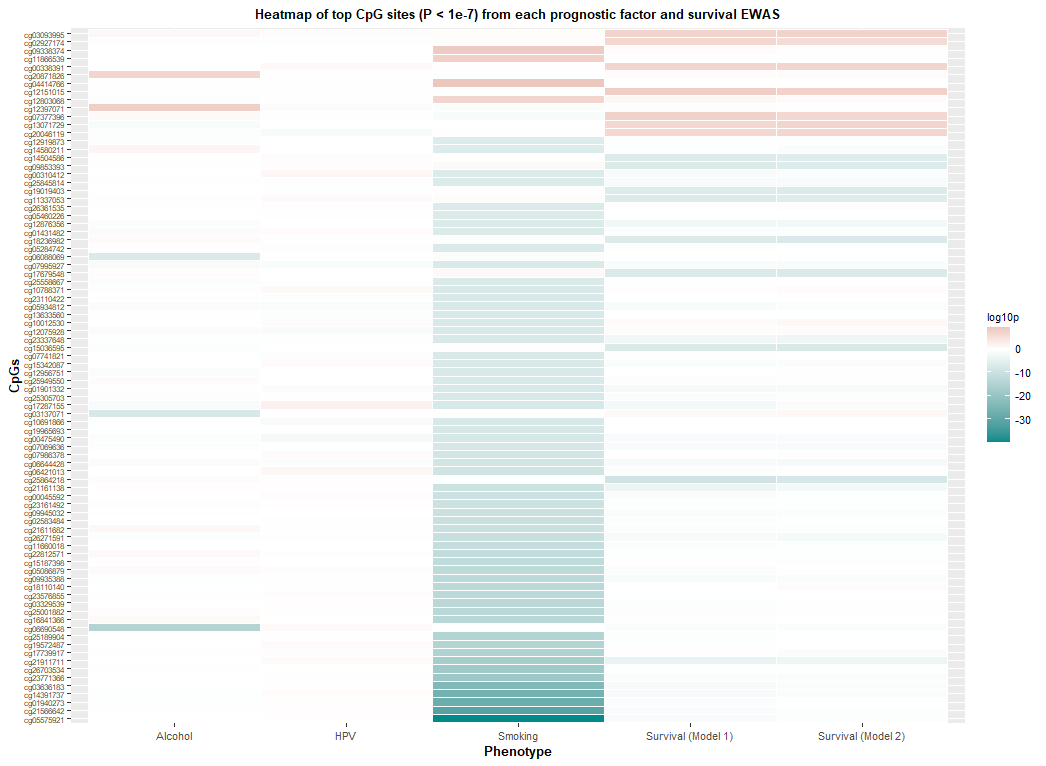
.

Supplementary Figure 1 - Heatmap showing correlation between top CpG sites (P<1x10^-7^) from each prognostic factor (alcohol consumption, HPV16 E6 seropositivity and smoking) and survival EWAS (Model 1: ~3-year survival adjusted for age sex and surrogate variables; Model 2: as Model 1, additionally adjusted for HPV16E6 seropositivity, smoking status and alcohol intake). Strength of association is shown by depth of colour; deeper red denotes a stronger phenotypic association with a hypermethylated CpG and deeper cyan denotes a stronger phenotypic association with a hypomethylated CpG

Supplementary Figure 2 - Surrogate variables correlated at P<0.05 (Pearson's) with laser surgery in HN5000


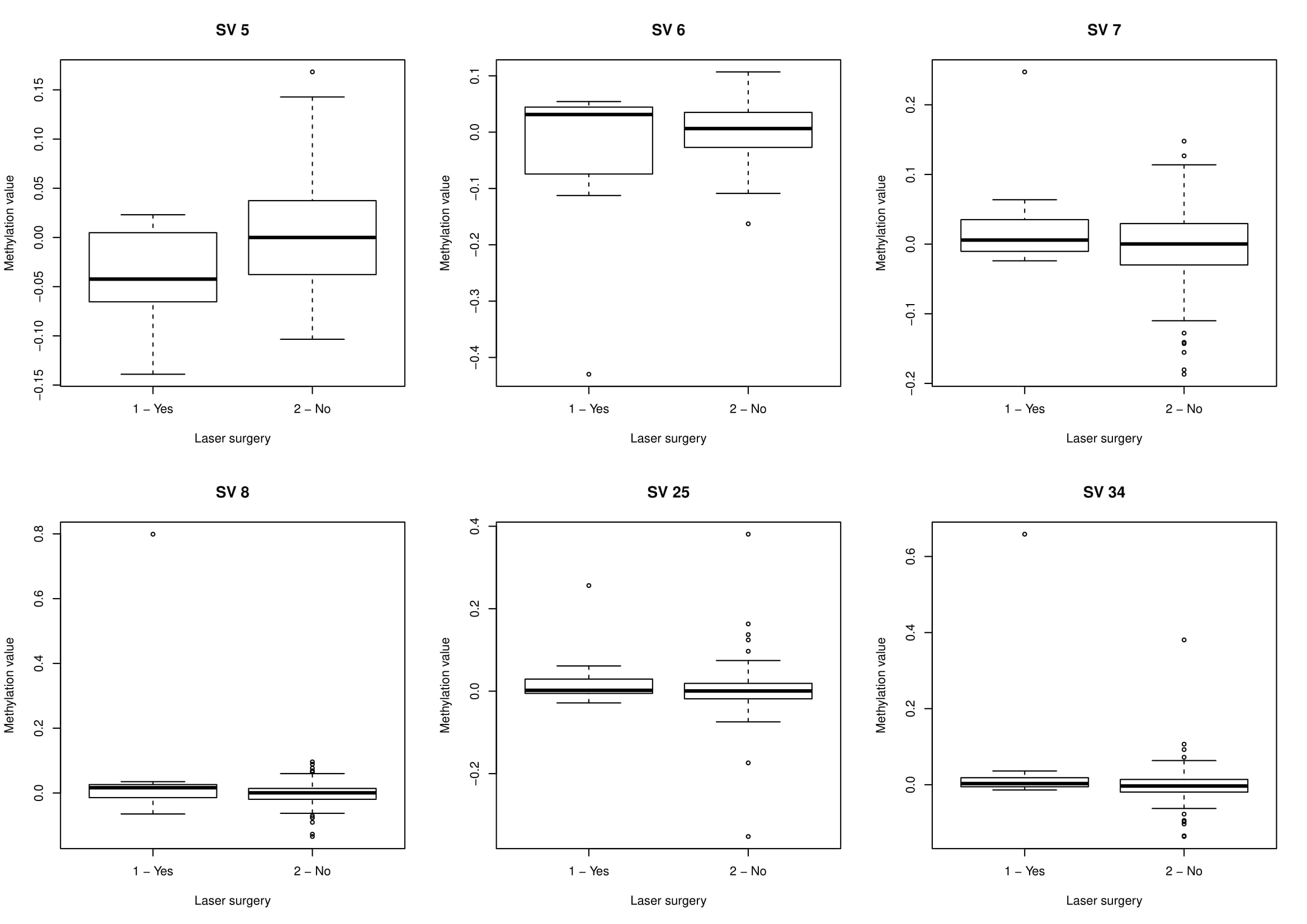


Supplementary Figure 3 - Surrogate variables correlated at P<0.05 (Pearson's) with surgery on an OPC primary tumour in HN5000


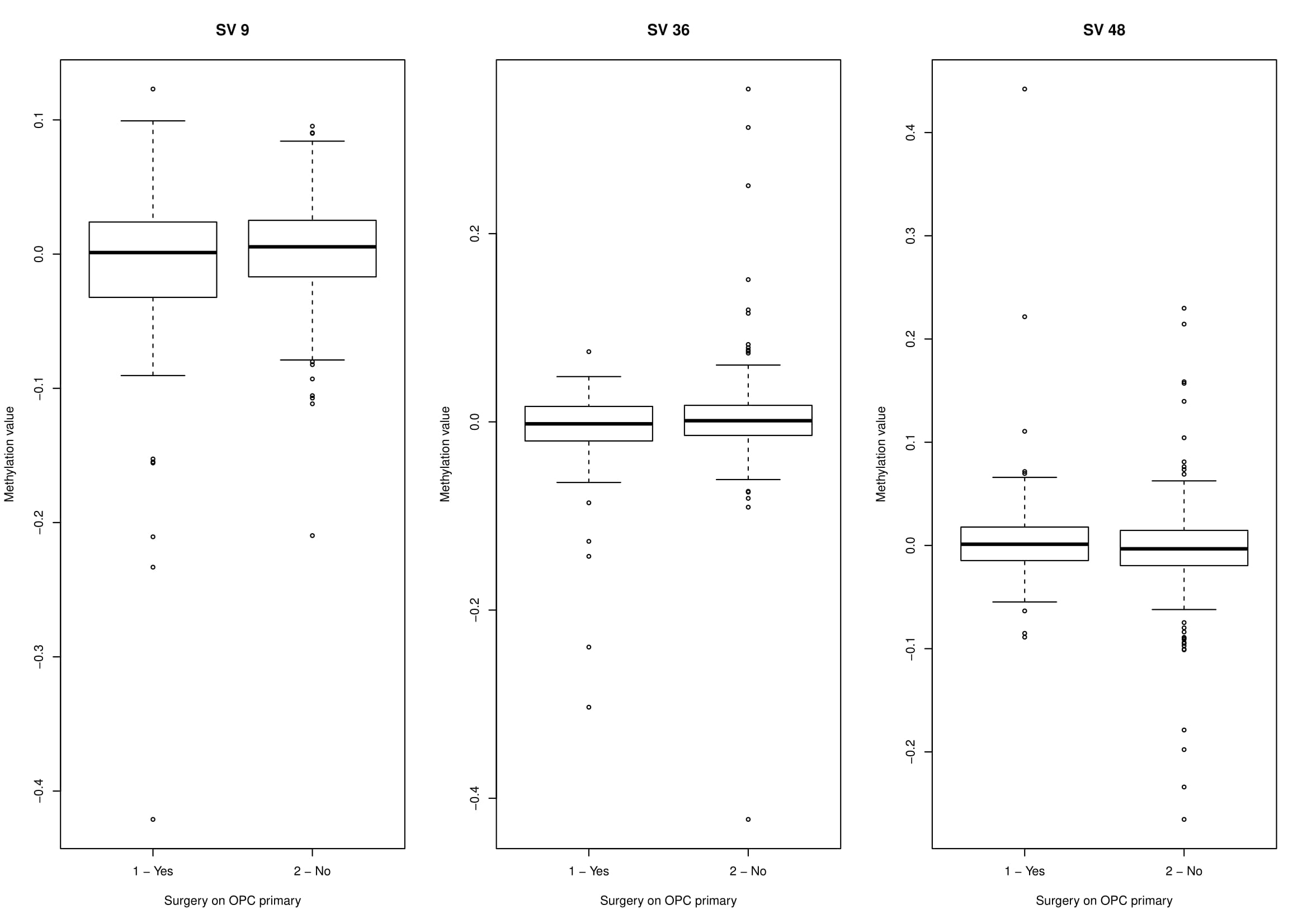


Supplementary Figure 4 - Surrogate variables correlated at P<0.05 (Pearson's) with neck resection surgery in HN5000


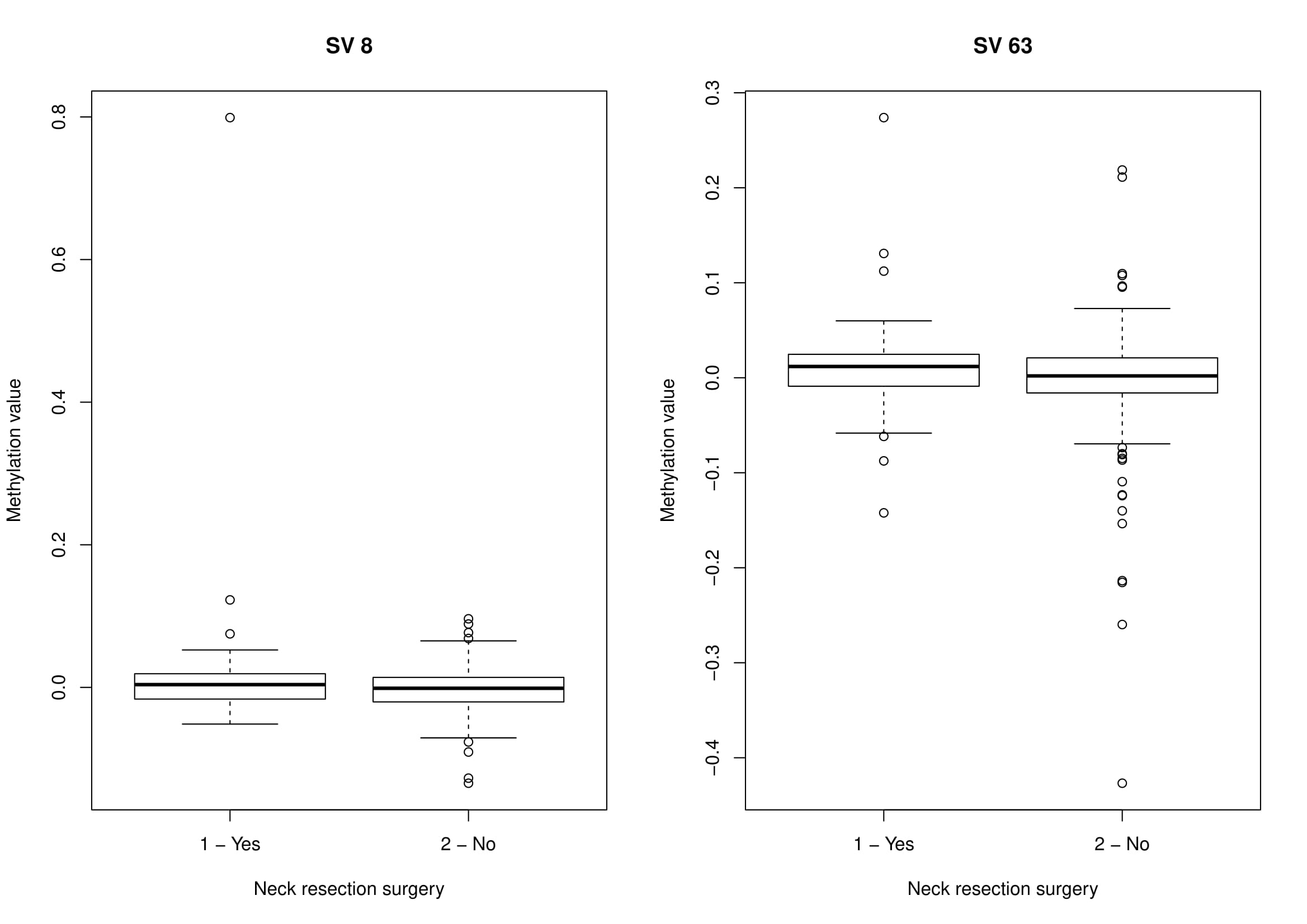


Supplementary Figure 5 - Surrogate variables correlated at P<0.05 (Pearson's) with teletherapy in HN5000


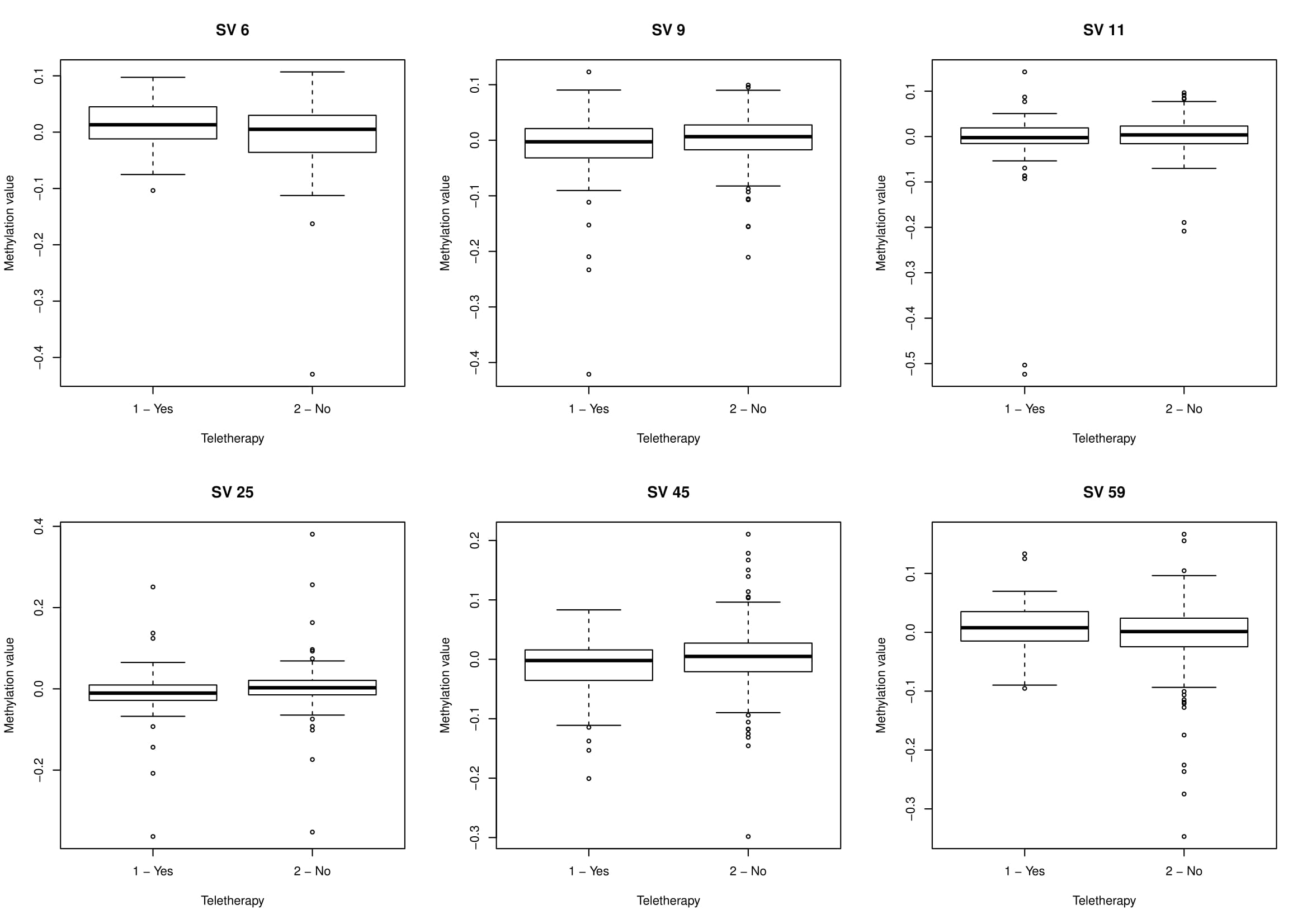


Supplementary Figure 6 - Surrogate variables correlated at P<0.05 (Pearson's) with chemotherapy in HN5000


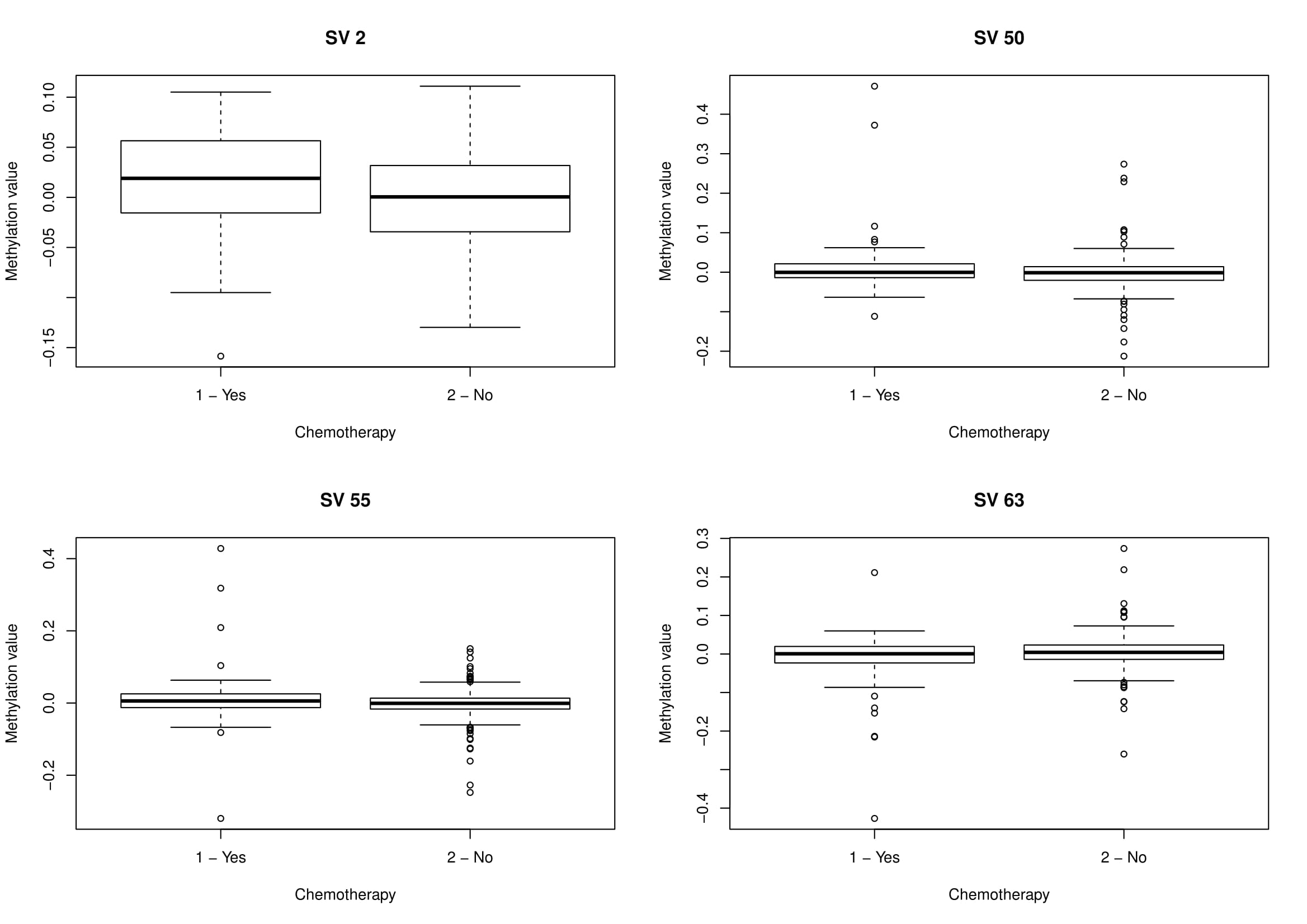


Supplementary Figure 7 - Surrogate variables correlated at P<0.05 (Pearson's) with chemoradiotherapy in HN5000


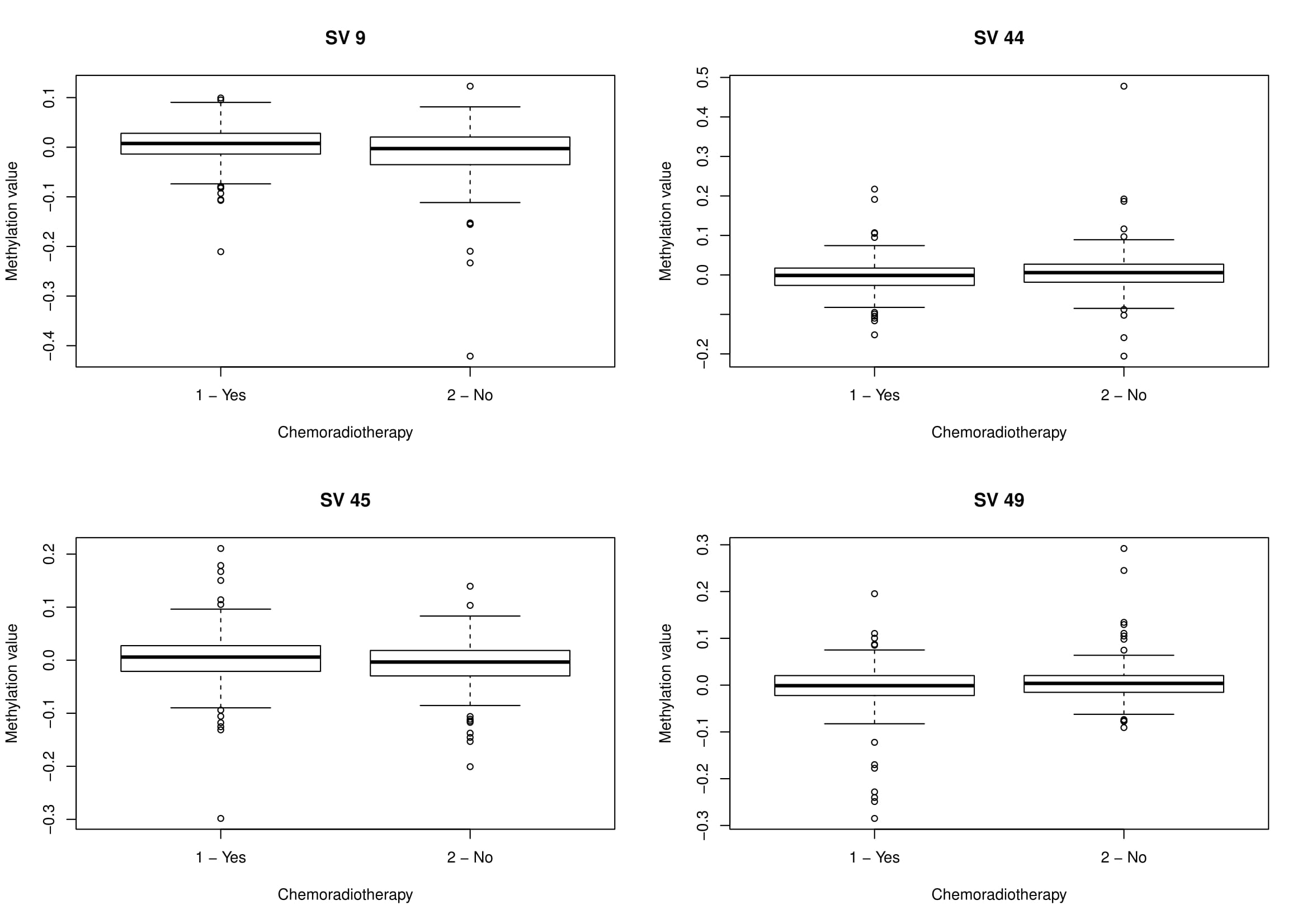


Supplementary Figure 8 - Surrogate variables correlated at P<0.05 (Pearson's) with TNM stage in HN5000


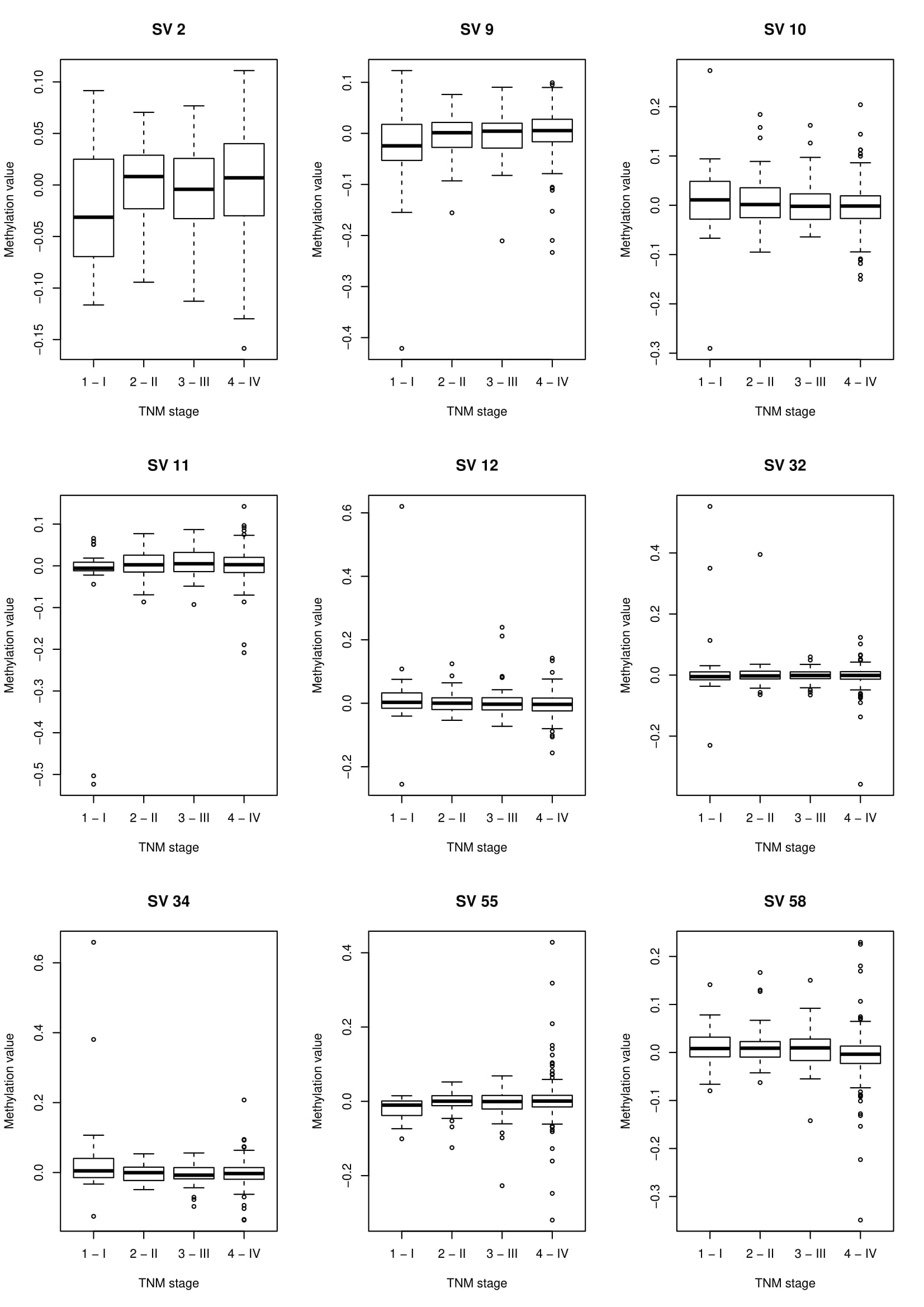


Supplementary Figure 9 - Surrogate variables correlated at P<0.05 (Pearson's) with neutrophil-to-lymphocyte ratio in HN5000


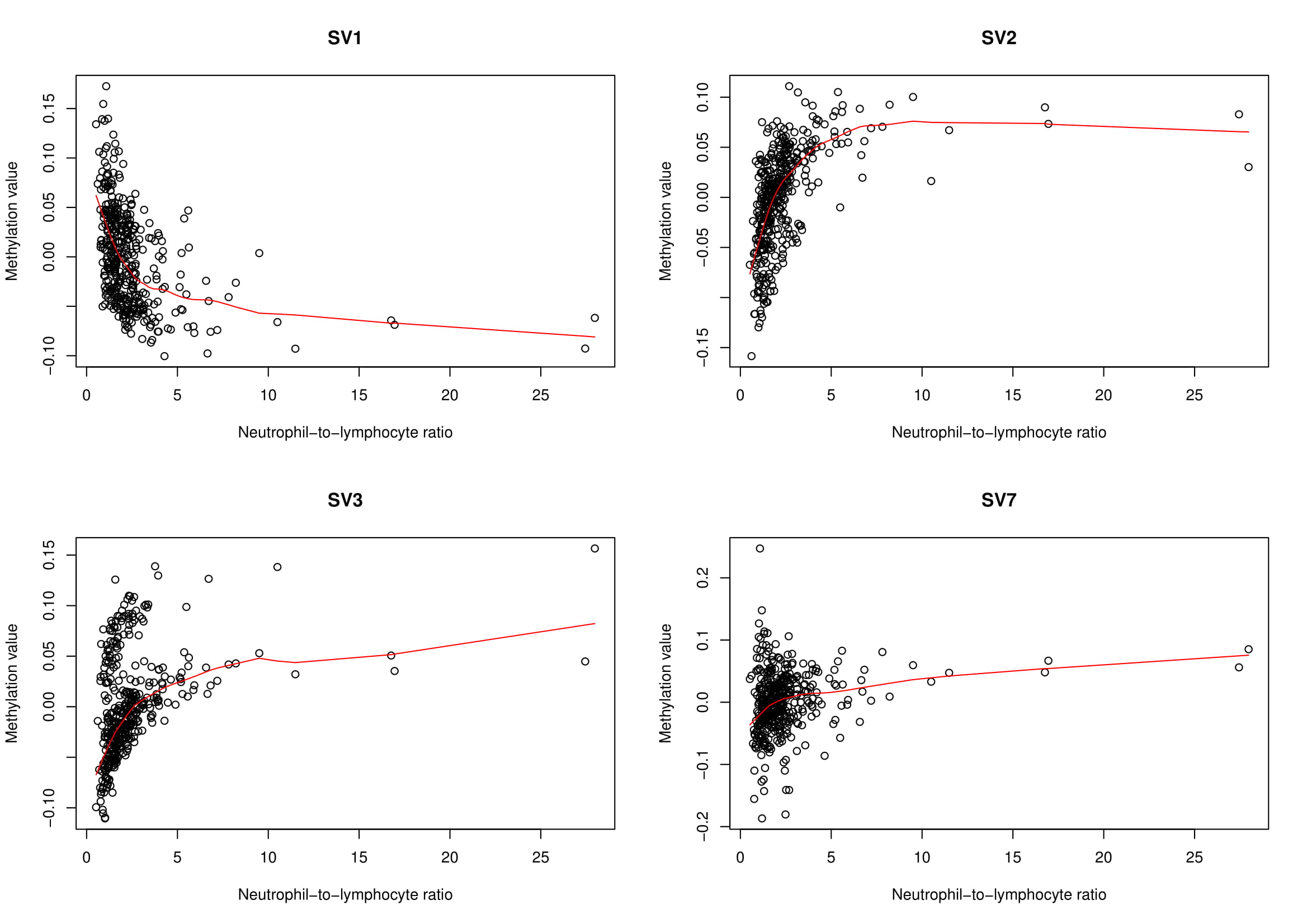

Supplement: Supplementary file 1 — Additional file 1:Supplementary Figure 1. Heatmap showing correlation between top CpG sites (P<1x10^-7^) from each prognostic factor (alcohol consumption, HPV16 E6 seropositivity and smoking) and survival EWAS (Model 1: ~3-year survival adjusted for age sex and surrogate variables; Model 2: as Model 1, additionally adjusted for HPV16E6 seropositivity, smoking status and alcohol intake). Strength of association is shown by depth of colour; deeper red denotes a stronger phenotypic association with a hypermethylated CpG and deeper cyan denotes a stronger phenotypic association with a hypomethylated CpG. Supplementary Figure 2. - Surrogate variables correlated at P<0.05 (Pearson's) with laser surgery in HN5000. Supplementary Figure 3. - Surrogate variables correlated at P<0.05 (Pearson's) with surgery on an OPC primary tumour in HN5000. Supplementary Figure 4. - Surrogate variables correlated at P<0.05 (Pearson's) with neck resection surgery in HN5000. Supplementary Figure 5. - Surrogate variables correlated at P<0.05 (Pearson's) with teletherapy in HN5000. Supplementary Figure 6. - Surrogate variables correlated at P<0.05 (Pearson's) with chemotherapy in HN5000. Supplementary Figure 7. - Surrogate variables correlated at P<0.05 (Pearson's) with chemoradiotherapy in HN5000. Supplementary Figure 8. - Surrogate variables correlated at P<0.05 (Pearson's) with TNM stage in HN5000. Supplementary Figure 9. - Surrogate variables correlated at P<0.05 (Pearson's) with neutrophil-to-lymphocyte ratio in HN5000. [file 13148_2020_870_MOESM1_ESM.docx]
